# Sociodemographic differences in discontinuation of high-risk medications: a retrospective cohort study

**DOI:** 10.1038/s41514-025-00310-4

**Published:** 2025-12-18

**Authors:** Katharina Tabea Jungo, Niteesh K. Choudhry, Julie C. Lauffenburger

**Affiliations:** 1 Center for Healthcare Delivery Sciences (C4HDS) and Division of Pharmacoepidemiology and Pharmacoeconomics, Department of Medicine, Brigham and Women’s Hospital and Harvard Medical School, Boston, MA USA; 2 Institute of Primary Health Care (BIHAM), University of Bern, Bern, Switzerland

**Keywords:** Diseases, Health care, Medical research, Risk factors

## Abstract

Older adults frequently use medications deemed high-risk, despite clinical recommendations supporting their discontinuation. We conducted a retrospective cohort study using administrative claims data from a large national U.S. health insurer (2017–2023) to assess high-risk medication discontinuation among adults aged ≥65 years and their association with sociodemographic factors. Among 729,705 long-term users of high-risk medications (mean age 74 [SD: 7], 59% female), only 22.8% discontinued for ≥90 consecutive days without subsequent refills (mean follow-up: 626 days). Discontinuation was more likely among Black adults (HR = 1.07, 95% CI: 1.03–1.11), particularly between 2020–2021, while men (HR = 0.89, 95% CI: 0.87–0.91) and those aged ≥75 years (HR = 0.86, 95% CI: 0.84–0.91) were less likely to discontinue compared to women and younger older adults, respectively. Notably, the combined effect of male gender and older age was associated with increased discontinuation (HR = 1.04, 95% CI: 1.02–1.06), whereas other sociodemographic combinations showed no meaningful interaction. When stratified by medication class, significant variation persisted for central nervous system, gastrointestinal, and pain medications but not for endocrine or cardiovascular medications. These findings highlight persistently low discontinuation rates and suggest the need for targeted interventions to reduce inappropriate medication use in older adults.

## Introduction

Older adults are at increased risk of inappropriate prescribing, which includes undertreatment (omission of necessary medications), overtreatment, and the use of potentially inappropriate medications^1,2^. Potentially inappropriate medication use in older adults is associated with various adverse events, including medication-related harm and increased healthcare costs^3–6^. When older adults use high-risk medications with risks outweighing benefits, guidelines (e.g., American Geriatrics Society Beers Criteria®^7^) recommend deprescribing^8^, defined as intentionally stopping or reducing these medications^6^. Implementing deprescribing is challenging, so we hypothesize that discontinuation rates of high-risk medications in routinely-collected data are low, though data on this remain limited^9,10^.

Previous deprescribing interventions have shown limited effectiveness^11^. One reason for the modest effectiveness may be that most deprescribing or medication optimization interventions have treated older adults as a homogeneous group, overlooking the need for diverse populations to have tailored deprescribing targets^12^. Patient characteristics, including gender and age, have been shown to influence the healthcare that older adults receive, including high-risk medications^12–18^. Furthermore, structural inequalities contribute to well-documented healthcare disparities in the United States^19,20^, including unequal access to prescription medications. White older adults are also more likely to receive overtreatment, not only with medications but also with other services (e.g., diagnostic testing)^12,21–23^.

Sociodemographic disparities in the overprescribing of high-risk medications have been examined in different studies^12,16,17,23^, whereas less is known about patterns of their discontinuation, particularly among long-term users. A few studies have explored sociodemographic differences in high-risk medication discontinuation (e.g., by age or race) but have focused on individual socioeconomic factors rather than considering their possible joint relationships^24–28^. Moreover, most prior studies focused on specific medication classes and few have used administrative claims data and instead relied on survey or electronic health record (EHR) data, often more prone to reporting and misclassification biases compared to claims^29–34^.

Therefore, we aimed to examine the discontinuation of a wide range of high-risk medications in older adults as well as the association between various sociodemographic factors and medication discontinuation.

## Results

The final cohort included 802,475 observations from 729,705 unique older adults, with each observation representing the initiation of a long-term high-risk medication. On average, individuals entered the cohort for 1 medication class (standard deviation (SD) = 0.3, median=1 (interquartile range (IQR) = 1–1). Their mean age was 74 years (SD = 7), and 59.2% were female (n = 432,140). Race was not recorded for 63.2% (460,801), and ethnicity was not recorded for 67.2% (n = 490,107). Sociodemographic and clinical characteristics by age, gender, and racial/ethnic groups are displayed in Table 1 and Table S3. There were more men and White older adults who had commercial health insurance, but characteristics were otherwise similar across groups.

**Table 1:** Selection of baseline characteristics^a^

Characteristics		By age, gender, ethnicity, and race; n (%)										
	All older adults	Age		Gender		Race				Ethnicity		
		65–74	≥75	Female	Male	Asian	Black	No database-recorded race	White	Hispanic	Non-Hispanic	No database-recorded ethnicity
n =	729,705^b^	424,299	305,406	432,140	297,565	12,777	39,020	460,801	217,107	40,977	198,621	490,107
Sociodemographic characteristics												
Age, mean (SD)	74.2 (6.7)	69.3 (2.7)	80.9 (4.3)	74.3 (6.8)	73.9 (6.5)	74.2 (6.7)	74.1 (6.7)	74.1 (6.7)	74.3 (6.7)	74.0 (6.6)	74.4 (6.8)	74.1 (6.7)
Product, n (%)												
Commercial	33,906 (4.6%)	27,903 (6.6%)	6,003 (2.0%)	15,523 (3.6%)	18,383 (6.2%)	646 (5.1%)	1927 (4.9%)	19,446 (4.2%)	11,886 (5.5%)	1991 (4.9%)	10,294 (5.2%)	21,620 (4.4%)
Medicare Advantage	695,799 (95.4%)	396,396 (93.4%)	299,403 (98.0%)	416,617 (96.4%)	279,182 (93.8%)	12,131 (94.9%)	37,093 (95.1%)	441,355 (95.8%)	205,221 (94.5%)	38,986 (95.1%)	188,327 (94.8%)	468,487 (95.6%)
Copay for all medications during the baseline period, mean (SD)	520.2 (860.6)	503.8 (864.0)	543.0 (855.3)	491.2 (792.0)	562.4 (949.8)	514.8 (810.6)	504.1 (877.9)	517.5 (868.3)	529.2 (843.7)	496.0 (856.2)	525.2 (859.5)	520.2 (861.3)
Copay for high-risk medication at index date, mean (SD)	9.1 (18.8)	8.8 (18.4)	9.5 (19.3)	9.2 (19.7)	8.9 (17.4)	9.0 (18.3)	8.5 (17.7)	9.0 (18.7)	9.4 (19.1)	8.6 (18.2)	9.2 (18.8)	9.1 (18.8)
Clinical characteristics												
Combined comorbidity score, mean (SD)	2.3 (3.0)	1.7 (2.8)	3.0 (3.2)	2.1 (2.9)	2.5 (3.2)	2.3 (3.0)	2.4 (3.1)	2.2 (3.0)	2.3 (3.0)	2.3 (3.0)	2.3 (3.1)	2.2 (3.0)
Mean number of medications filled (SD)	13.2 (6.7)	13.1 (6.9)	13.4 (6.4)	13.5 (6.8)	12.8 (6.5)	13.1 (6.6)	13.5 (6.8)	13.2 (6.7)	13.2 (6.7)	13.4 (6.8)	13.3 (6.7)	13.2 (6.7)
Mean number of days hospitalized (SD)	3.1 (9.6)	2.6 (9.6)	3.8 (9.7)	2.8 (8.9)	3.5 (10.6)	3.2 (10.0)	3.4 (10.3)	3.1 (9.6)	3.2 (9.6)	3.1 (10.0)	3.2 (9.7)	3.1 (9.6)
Mean number of outpatient visits (SD)	31.9 (32.8)	28.7 (30.7)	36.3 (35.1)	31.8 (32.0)	32.0 (34.1)	31.2 (32.6)	33.1 (34.6)	31.6 (32.8)	32.3 (32.6)	31.8 (33.3)	32.5 (33.0)	31.7 (32.7)
Mean claims-based Frailty Index (SD)	0.2 (0.1)	0.2 (0.1)	0.2 (0.1)	0.2 (0.1)	0.2 (0.1)	0.2 (0.1)	0.2 (0.1)	0.2 (0.1)	0.2 (0.1)	0.2 (0.1)	0.2 (0.1)	0.2 (0.1)

There was no missing data.

^a^The rest of the baseline characteristics can be found in Table S3 in the Supporting Material.

^b^This table is restricted to older adults’ first cohort entry.

Individuals were followed for an average of 626 days (SD = 561; median = 440, IQR = 192–894 days) until discontinuation or censoring. Table S2 shows the censoring reasons by age category, gender, race and ethnicity. During follow-up, 12.7% of older adults (n = 101,789) died, with similar rates across race and ethnicity groups.

In total, 22.6% (n = 181,112) of all high-risk medications were discontinued over follow-up (Table 2). Examining medication discontinuation at the patient level was consistent: 22.8% (n = 166,692) of older adults discontinued ≥1 high-risk medication. Across medication categories, the discontinuation was lowest for cardiovascular (14.7%) and highest for pain medications (29.4%) (Table S4).

**Table 2:** Discontinuation vs continuation by age, gender, ethnicity, and race category

		Number of observations stratified by whether the high-risk medication use was discontinued (n = 802,475)^a^	
		Continuation	Discontinuation
		621,363 (77.4%)	181,112 (22.6%)
Age	65 to 74 years	350,567 (75.4%)	114,631 (24.6%)
	≥75 years	270,796 (80.3%)	66,481 (19.7%)
Gender	Female	365,659 (76.5%)	112,096 (23.5%)
	Male	255,704 (78.8%)	69,016 (21.3%)
Race	Asian	10,759 (76.7%)	3,274 (23.3%)
	Black	33,003 (77.1%)	9,833 (22.9%)
	No database-recorded race	392,595 (77.4%)	114,622 (22.6%)
	White	185,006 (77.6%)	53,383 (22.4%)
Ethnicity	Hispanic	34,379 (76.5%)	10,570 (23.5%)
	Non-Hispanic	169,286 (77.6%)	48,807 (22.4%)
	No database-recorded ethnicity	417,698 (77.4%)	121,735 (22.6%)

^a^802,475 observations from 729,705 unique individuals. Among these unique individuals, 166,613 (22.8%) discontinued at least one high-risk medication.

In adjusted models without interaction terms, compared to White older adults, Black older adults were more likely to discontinue their long-term high-risk medication (hazard ratio (HR) = 1.04, 95%CI 1.01–1.06); discontinuation in this context is considered beneficial based on clinical guidelines. Older adults with Hispanic ethnicity also were more likely to discontinue (HR = 1.05, 95%CI 1.02–1.07). By contrast, men (HR = 0.91, 95%CI 0.90–0.92) and adults aged ≥75 years (HR = 0.88, 95%CI 0.87–0.89) were less likely to discontinue compared to women or adults aged 65–74 years, respectively (Table S5). In addition, those insured by Medicare Advantage (vs. commercial insurance) (HR = 1.22, 95%CI 1.19–1.25), filling more medications at baseline (HR = 1.01, 95%CI 1.01–1.01) and having liver disease (HR = 1.08, 95%CI 1.06–1.10) were more likely to discontinue. By contrast, patients with some comorbidities (e.g., dementia [HR = 0.73, 95%CI 0.71–0.76], psychosis [HR = 0.86, 95%CI 0.82–0.90]) were less likely to discontinue (full list of variables shown in Table S5).

**Fig. 1:**
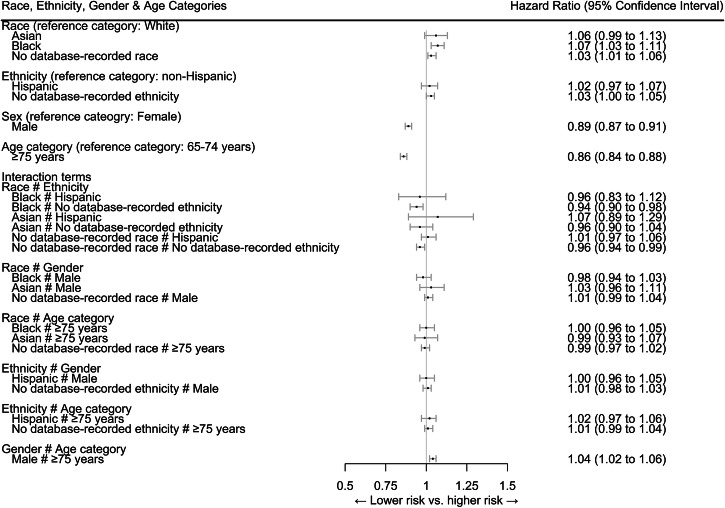
Association between age, gender, ethnicity, and race and the discontinuation of high-risk medication (Cox regression model^1^, n = 802,475). ^1^Model adjusted for the following baseline characteristics: type of healthcare insurance, copay for medications during the baseline period, copay for the high-risk medication at the index date, the Combined Comorbidity Score, comorbidities (as measured for Combined Comorbidity Score: congestive heart failure, dementia, renal failure, weight loss, hemiplegia, alcohol abuse, any tumor, metastatic cancer, cardiac arrhythmias, chronic pulmonary disease, coagulopathy, complicated diabetes, deficiency anemias, fluid and electrolyte disorders, liver disease, peripheral vascular disorder, psychosis, pulmonary circulation disorders, HIV/AIDS, hypertension), number of medications filled, number of days hospitalized, number of outpatient visits, claims-based frailty index, and the year of cohort entry. The complete table can be found in Table S5 in the supporting material.

In adjusted models with interaction terms, compared to White older adults, Black (HR = 1.07, 95%CI 1.03–1.11) older adults were more likely to discontinue their long-term high-risk medication. Older adults with no database-recorded ethnicity also showed a higher likelihood of discontinuation but this was not statistically significant after Bonferroni correction. Men (HR = 0.89, 95%CI 0.87–0.91) and adults aged ≥75 years (HR = 0.88, 95%CI 0.84–0.88) were less likely to discontinue high-risk medication compared to women or adults aged 65–74 years, respectively (Fig. 1). The associations between other patient characteristics and discontinuation remained consistent across models with and without interaction terms. While male gender and age ≥75 years were each individually associated with a lower likelihood of discontinuation, their combined presence was associated with a 4% higher likelihood (HR = 1.04, 95% CI: 1.02–1.06), a deviation from the expected combined effect. No other combinations of sociodemographic characteristics were associated with changes in discontinuation (Table S5).

All sensitivity and subgroup analyses were consistent for age and gender except for the smaller 2023 subgroup where the associations were no longer significant (Table S6). However, there were some key differences for race and ethnicity. Black race was no longer associated with being more likely to discontinue in patients using <5 medications and when having follow-up time capped at 365 days. Similarly, there was variations by calendar year, with Black race only being significantly associated with higher discontinuation in 2020 and 2021.

Like the overall results, the same trends across age were observed for central nervous system medications (HR = 0.89, 95%CI 0.94–0.94), anticholinergic medications (HR = 0.89, 95%CI 0.83–0.94), gastrointestinal medications (HR = 0.87, 95%CI 0.84–0.90), and pain medications (HR = 0.88, 95%CI 0.84–0.92) when examined individually, but not for other medication categories (Table S7). The same trends across gender were observed for gastrointestinal (HR = 0.92, 95%CI 0.88–0.95) and pain medications (HR = 0.85, 95%CI 0.81–0.88). In analyses by medication categories, we did not observe evidence for an association between race and discontinuation.

## Discussion

In this nationwide retrospective cohort study, less than 1 in 4 older adults discontinued high-risk medication after becoming long-term users, showing the need for effective medication optimization strategies. Individually, male gender and age ≥75 were each linked to a lower likelihood of high-risk medication discontinuation; however, their combination was associated a higher likelihood of discontinuation contrary to what would be expected from their separate effects. By contrast, Black older adults were more likely to discontinue high-risk medications, but this was primarily driven by 2020 and 2021, or COVID-19 pandemic years. We also found sociodemographic differences in central nervous system, gastrointestinal, and pain medications, consistent with the main findings, but no significant differences for endocrine, or cardiovascular medications.

Measuring medication discontinuation in real-world data is challenging^35–37^. Previous studies reported discontinuation rates ranging from 6% to 49%, depending on the discontinuation definitions used (e.g., length of gap, no subsequent fills) and medication types studied^35,36,38^. Compared to other claims-based studies, we used a more conservative definition now recommended^35^. Claims data lack information on the reasons for discontinuation, so we cannot rule out non-adherence instead of intentional deprescribing by healthcare professionals. This is why we intentionally used “discontinuation” rather than “deprescribing” throughout.

Comparison with previous literature is challenging due to differences in discontinuation definitions study populations (e.g., older adults with dementia or in nursing homes), focus on specific medications, lack of race/ethnicity information, and use of survey or EHR data^31,35,36,38–40^. Our finding that adults aged ≥75 were less likely to discontinue high-risk medication aligns with other analyses of claims data^40^, though some studies report opposite results in the associations with race/ethnicity and discontinuation (e.g., acetylcholinesterase inhibitors (AChEIs), bisphosphonates)^24,38,39^ but often focused on older adults with dementia or in nursing homes. In contrast to our findings, in an analysis of EHR data limited to prescribing data, higher age was associated with a lower likelihood of continuing benzodiazepine receptor agonists in adults aged ≥65 years^31^. Comparability is limited due to varying age cutoffs for ‘younger’ vs. ‘older’ adults, though previous studies suggest less persistence in younger older adults, which may help explain our findings^41,42^. Others also found differences in discontinuation likelihood across comorbidities (e.g., dementia, cancer)^24,38^, but associations varied across studies, which were conducted in narrower study populations (e.g., nursing home residents and patients with depression).

The lower likelihood of male older adults discontinuing long-term high-risk medications merits further attention. This finding is in line with an EHR data analysis of adults aged ≥75 years from Belgium that found higher odds of discontinuation for women^29^. However, a longitudinal survey study of nursing home residents in Europe and Israel showed a lower likelihood of discontinuation in women^30^, which could be explained by the different data sources (EHR vs. claims). The gender difference in our findings may reflect lower adherence to long-term medication in women, due to barriers such as access and other systemic challenges in obtaining treatment^42,43^.

Considering known systematic barriers in access to healthcare, the observed sociodemographic differences warrant further discussion. Comparisons with existing literature, particularly for evidence-based medications, indicate well-documented gender, racial and ethnic differences in adherence and persistence, for example, to cardiovascular or antidementia medication^26–28^. These studies show that White and male older adults are less likely to have adherence issues. This discrepancy reflects broader inequities in access to care that could spill over into increased adherence and persistence to all types of treatments for White patients^42,44,45^. By conditioning our cohort on long-term users across all sociodemographic groups, we were able to observe the isolated association with discontinuation. For example, Black older adults were more likely than White older adults to discontinue high-risk medications during the COVID-19 pandemic’s first two years, likely due to widespread challenges in healthcare access during this period that were even worse than other years^46,47^. The association with not having a database-recorded ethnicity is more challenging to interpret, but is likely due to historically less reporting by non-White individuals^48^.

In recent years, there has been growing interest in interventions to optimize high-risk medication discontinuation in older adults^49^. However, previous interventions have often shown modest effectiveness, perhaps in part because they have used a one-size-fits-all approach^50–52^. Our findings show that there may be a set of variables, including sociodemographic and clinical characteristics, that may affect the likelihood of discontinuation. Although our findings do not offer a specific solution, they underscore the need to test the tailoring of deprescribing interventions - both in terms of delivery and content - according to patient sociodemographic characteristics and medication classes, in further research. Finally, while medication reviews should be routine for all older adults, racial and ethnic minorities are less likely to receive them in practice, due to factors related to healthcare access, such as fewer primary care visits^53–55^. However, it is promising that once older adults receive a medication review, there is no evidence for a differential impact on inappropriate use by race or ethnicity^56^.

This research was strengthened by using self-reported race and ethnicity^48,57^, but has several limitations. First, some individuals had no database-recorded race and ethnicity data; rather than excluding them we included them for generalizability^48^. Second, these data contain older adults with private health insurance or Medicare Advantage, limiting generalizability. Third, claims data may not fully capture medication use, but we used conservative definitions of long-term use and discontinuation-combining fills and days’ supply and requiring no subsequent fills - to minimize misclassification. A 90-day gap for defining discontinuation had good specificity^36^, and requiring no further fills helps exclude false discontinuations^58,59^. Our discontinuation definition excluded partial dose reductions which may be beneficial but still not fully guideline concordant. Similarly, claims data cannot confirm the intentionality of discontinuation (i.e., patient or provider directed). We focused on new long-term users, therefore, new users who discontinued high-risk medications shortly after initiation were likely excluded from our analyses. Lastly, although we relied on the widely recognized American Geriatrics Society Beers Criteria® and the Medicines Scorecard of the Anticholinergic Burden Calculator (ACB calculator) to identify high-risk medications and focused on those with the strongest evidence and highest risk regardless of comorbidity, these criteria are criterion-based rather than judgment-based^7,60^. Therefore, they may have misclassified certain medications as potentially inappropriate, even when their use was clinically justified, and efforts to reduce their use may not be appropriate in every case, possibly resulting in undertreatment contrary to indication. Furthermore, the potential risks vary across the 16 categories of high-risk medications, for example, between proton pump inhibitors and central nervous system drugs.

In conclusion, once older adults begin long-term use of high-risk medication, less than 1 in 4 discontinue it. This shows the need for effective deprescribing interventions to reduce high-risk medication use in older adults. Men and adults aged ≥75 years were less likely to discontinue high-risk medication, though male adults aged ≥75 years were more likely to discontinue. Black older adults were more likely to discontinue the high-risk medication, though this was heavily influenced by the COVID-19 pandemic and widespread challenges in healthcare access to medications and visits during this period.

## Methods

### Study design and data source

In this retrospective cohort study, approved by the Institutional Review Board of Brigham and Women’s Hospital, we used administrative claims from Optum, a major health insurance provider in the United States of America. Optum’s de-identified Clinformatics® Data Mart Database (CDM or Clinformatics®) is derived from a database of administrative health claims for members of large commercial and Medicare Advantage health plans. Clinformatics® utilizes medical and pharmacy claims to derive patient-level enrollment information, health care costs, and resource utilization information. The population is geographically diverse, spanning all 50 states and is de-identified under the Expert Determination method consistent with HIPAA and managed according to Optum® customer data use agreements. CDM administrative claims submitted for payment by providers and pharmacies are verified, adjudicated and de-identified prior to inclusion. Commercial private health insurance plans are available from both for-profit and nonprofit organizations^61^. Medicare Advantage, also known as Medicare Part C, is an alternative to traditional Medicare offered by private insurance companies approved by Medicare^62^. We followed the Strengthening the Reporting of Observational Studies in Epidemiology (STROBE) reporting guidelines^63^. All research was performed in accordance with the Declaration of Helsinki^64^.

### Definition of high-risk medication use

We identified medications with a moderate or high quality of evidence and a strong level of recommendation for avoidance in older adults from the American Geriatric Society Beers Criteria® (2019 version) and medications with strong anticholinergic properties from the widely-used Medicines Scorecard of the Anticholinergic Burden Calculator (ACB calculator)^7,60^. We selected medications from 16 different medication classes for which potential harms are deemed to outweigh benefits in older adults (Table S1). We excluded medications that were primarily available over the counter (OTC) (i.e., aspirin, ibuprofen, ketoprofen, naproxen) or required information unavailable in claims data (e.g., lab values) to consider appropriateness. Molecularly-related medications were considered interchangeable (e.g., benzodiazepines), so we measured use at the class level.

### Cohort definition

**Fig. 2:**
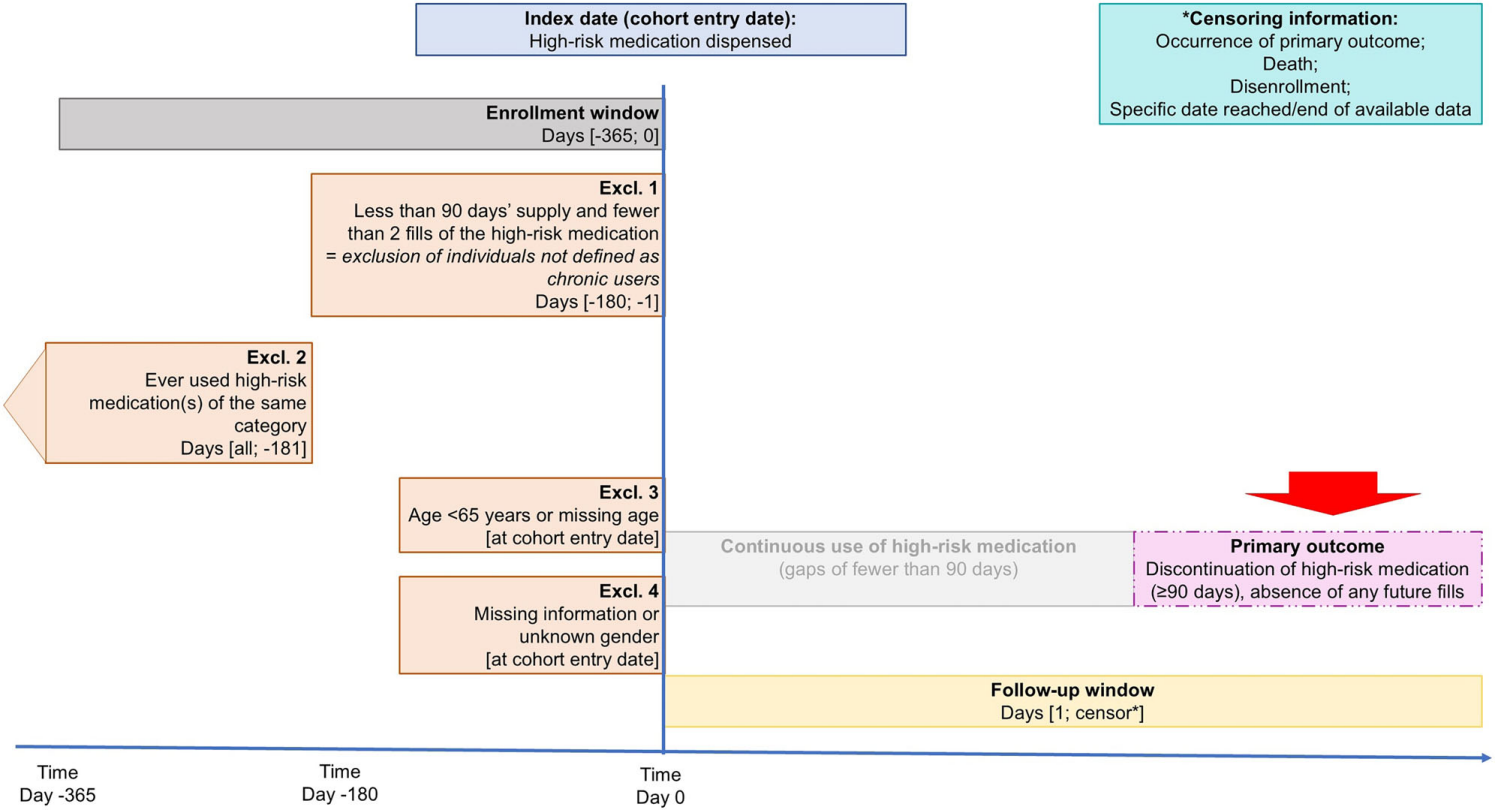
Visualization of inclusion and exclusion criteria.

We constructed a cohort of relatively new long-term users of high-risk medications; we did this to mitigate survivor bias and other confounding related to potential differences in length of insurance enrollment. The index date was defined as the date on which older adults filled a high-risk medication. Thus, to be included, older adults had to: (i) start high-risk medication between 2017–2023 (“index date”), (ii) maintain continuous enrollment in the 365 days prior to the index date^12,65^, (iii) be long-term users (≥90 days’ supply^12,65,66^ and ≥2 refills in the 180 days prior^12^), (iv) recently start use (no use until 181 days prior to index date), (v) be ≥65 years old with no missing age data, and (vi) have gender recorded (Fig. 2, study flow chart Figure S1). All cohorts were generated with the Aetion Evidence Platform^67^. All individuals were followed until censored, including for outcome occurrence, death, disenrollment, or end of data availability.

### Exposure

We measured age, gender, ethnicity, and race from enrollment files, which were self-reported by patients. For age, we stratified between 65–74 years and ≥75 years to distinguish between the “younger” older adults, who generally have better health, need minimal daily living support, and are often still active in the US job market^68–70^. Rather than excluding them, to ensure generalizability, those for whom race and ethnicity information was not reported were included and classified as “no database-recorded race/ethnicity”.

### Outcome

The outcome was discontinuation of high-risk medications, defined as the start of continuous non-occurrence of this medication of ≥90 days and the absence of any future fills. The continuous non-occurrence had to start within a 90-day grace period following the end of continuous use (allowing gaps of <90 days). We defined the discontinuation date as the 91^st^ day after the start of the supply gap, a more conservative approach than using the gap’s first day^38^. A ≥ 90-day gap has been shown to accurately capture discontinuation, and our definition requiring no subsequent fills has been shown to more likely reflect true discontinuations^35,36,38,59^. Of note, as with all types of claims data, it is not possible to distinguish clinician versus patient-directed discontinuation, similar to other discontinuation studies^71,72^.

### Covariates

We assessed patient characteristics as covariates in the 365 days prior to the index date, including the following: comorbidities and comorbidity score as measured by the Combined Comorbidity Score^73,74^, number of unique generic medications filled, healthcare utilization (number of inpatient visits and number of outpatient visits), claims-based Frailty Index^75–77^, type of health insurance (commercial vs. Medicare Advantage), copayments for all medications during the baseline period, and index date copayment for the high-risk medication The Combined Comorbidity Score ranges from -2 to 18, with higher scores indicating a greater burden of comorbid conditions^73,74^. The claims-based Frailty Index (range: 0–1) uses administrative data to assess a patient’s level of frailty, with higher scores indicating more advanced frailty^75–77^.

### Statistical analysis

We first described patient characteristics by race, ethnicity, gender, and age. For older adults entering the cohort for multiple medication classes, baseline characteristics were analyzed at their first entry. Discontinuation rates were then described overall and by subgroups. After testing the proportional hazards assumption and concluding that the conditions were met, we used Cox regression models to estimate the association between sociodemographic factors and time until the discontinuation of high-risk medication. Shorter time to discontinuation and lower hazard ratios would be clinically preferable, indicating shorter use of high-risk medications. The models were adjusted for all measured baseline characteristics and clustering at the patient level. To assess the robustness of main effects, we added interaction terms between race, ethnicity, gender, and age.

In secondary analyses, we used Cox regression models by individual medication category (anticholinergic, cardiovascular, central nervous system, endocrine, gastrointestinal, and pain medications, grouped like in Table 2 of the American Geriatric Society Beers Criteria®^7^).

In subgroup analyses, we stratified the Cox regression model by the number of medications used at baseline (absence of polypharmacy [<5 medications] vs. presence of polypharmacy [≥5 medications]^78^) and by cohort entry year (e.g., 2017, 2018). In sensitivity analyses, we repeated the analyses by restricting to individuals having ≥1 ambulatory visit during the baseline period, capping the follow-up period at 365 days, restricting to individuals’ first cohort entry (i.e., first medication), and focusing on American Geriatric Society Beers Criteria® only. We also ran an accelerated failure time (AFT) model, an alternative modeling approach, to test the findings’ robustness. We repeated our main analyses using a cohort where new long-term use at baseline was defined more strictly, requiring a proportion of days covered (PDC) of ≥80% in the 365 days prior to the index date^79^.

Statistical analyses were conducted in Stata (v17). Forest plots were created in R (v4.3.1). All p-values were two-sided, with significance levels adjusted using the Bonferroni method for multiple comparisons.

### Ethical approval

This project was approved by the Institutional Review Board of Brigham and Women’s Hospital (2023P000438). All research was performed in accordance with the Declaration of Helsinki^64^.

## Supplementary information

2025-10-31_supporting material_v1_clean.

## Data Availability

No additional data available.
