# Supplementary material for: Sociodemographic differences in discontinuation of high-risk medications: a retrospective cohort study
Source: NPJ Aging. 2025 Dec 18;12(1):13. doi: 10.1038/s41514-025-00310-4 (PMC12820038; doi:10.1038/s41514-025-00310-4)
Supplement: Supplementary file 1 — 2025-10-31_supporting material_v1_clean. [file 41514_2025_310_MOESM1_ESM.docx]

Supporting Material

Sociodemographic differences in discontinuation of high-risk medications: a retrospective cohort study

Katharina Tabea Jungo^1,2^, Niteesh K. Choudhry^1^, Julie C. Lauffenburger^1^

^1^ Center for Healthcare Delivery Sciences (C4HDS) and Division of Pharmacoepidemiology and Pharmacoeconomics, Department of Medicine, Brigham and Women's Hospital and Harvard Medical School, Boston, Massachusetts, USA

^2^ Institute of Primary Health Care (BIHAM), University of Bern, Bern, Switzerland

Table of content

[Table S1. Table of high-risk medications included 2](#_Toc212815661)

[Table S2. Censoring events, by age, gender, ethnicity, and race category 4](#_Toc212815662)

[Figure S1. Cohort definition flow chart 5](#_Toc212815663)

[Table S3. Continuation of baseline characteristics 8](#_Toc212815664)

[Table S4. Discontinuation vs. continuation by medication type 9](#_Toc212815665)

[Table S5. Complete results for the association of age, gender, ethnicity, and race and the discontinuation of high-risk medications (Cox regression model) 9](#_Toc212815666)

[Table S6. Association of age, gender, ethnicity, and race and the discontinuation of high-risk medications (Cox regression model): Analyses without interaction terms, subgroup and sensitivity analyses 11](#_Toc212815667)

[Table S7. Association of age, gender, ethnicity, and race and the discontinuation of high-risk medications (Cox regression model): By medication class 13](#_Toc212815668)

Table S1. Table of high-risk medications included

| **Overall medication category**  **(grouped)** | **Organ system or pharmacology** | **Medication classes** | **Generic drug name** |
| --- | --- | --- | --- |
| **Anticholinergic medications** | Anticholinergics | Antispasmodics | Clidinium-chlordiazepoxide |
| **Anticholinergic medications** | Anticholinergics | Antispasmodics | Hyoscyamine |
| **Anticholinergic medications** | Anticholinergics | First-generation antihistamines | Brompheniramine |
| **Anticholinergic medications** | Anticholinergics | First-generation antihistamines | Carbinoxamine |
| **Anticholinergic medications** | Anticholinergics | First-generation antihistamines | Chlorpheniramine |
| **Anticholinergic medications** | Anticholinergics | First-generation antihistamines | Clemastine |
| **Anticholinergic medications** | Anticholinergics | First-generation antihistamines | Cyproheptadine |
| **Anticholinergic medications** | Anticholinergics | First-generation antihistamines | Dexbrompheniramine |
| **Anticholinergic medications** | Anticholinergics | First-generation antihistamines | Dexchlorpheniramine |
| **Anticholinergic medications** | Anticholinergics | First-generation antihistamines | Dimenhydrinate |
| **Anticholinergic medications** | Anticholinergics | First-generation antihistamines | Diphenhydramine |
| **Anticholinergic medications** | Anticholinergics | First-generation antihistamines | Doxylamine |
| **Anticholinergic medications** | Anticholinergics | First-generation antihistamines | Hydroxyzine |
| **Anticholinergic medications** | Anticholinergics | First-generation antihistamines | Meclizine |
| **Anticholinergic medications** | Anticholinergics | First-generation antihistamines | Promethazine |
| **Anticholinergic medications** | Anticholinergics | First-generation antihistamines | Pyrilamine |
| **Anticholinergic medications** | Anticholinergics | First-generation antihistamines | Triprolidine |
| **Anticholinergic medications** | Antimuscarinics | *not specified* | Darifenacin |
| **Anticholinergic medications** | Antimuscarinics | *not specified* | Fesoterodine |
| **Anticholinergic medications** | Antimuscarinics | *not specified* | Flavoxate |
| **Anticholinergic medications** | Antimuscarinics | *not specified* | Hyoscine hydrobromide |
| **Anticholinergic medications** | Antimuscarinics | *not specified* | Oxybutynin |
| **Anticholinergic medications** | Antimuscarinics | *not specified* | Propiverine |
| **Anticholinergic medications** | Antimuscarinics | *not specified* | Solifenacin |
| **Anticholinergic medications** | Antimuscarinics | *not specified* | Tolterodine |
| **Anticholinergic medications** | Antimuscarinics | *not specified* | Trospium |
| **Cardiovascular medications** | Antithrombotics | *not specified* | Dipyridamole |
| **Cardiovascular medications** | Cardiovascular | Peripheral alpha-1 blockers | Doxazosin |
| **Cardiovascular medications** | Cardiovascular | Peripheral alpha-1 blockers | Prazosin |
| **Cardiovascular medications** | Cardiovascular | Peripheral alpha-1 blockers | Terazosin |
| **Central nervous system medications** | Central nervous system | Antidepressants | Amitriptyline |
| **Central nervous system medications** | Central nervous system | Antidepressants | Amoxapine |
| **Central nervous system medications** | Central nervous system | Antidepressants | Clomipramine |
| **Central nervous system medications** | Central nervous system | Antidepressants | Desipramine |
| **Central nervous system medications** | Central nervous system | Antidepressants | Doxepin |
| **Central nervous system medications** | Central nervous system | Antidepressants | Imipramine |
| **Central nervous system medications** | Central nervous system | Antidepressants | Nortriptyline |
| **Central nervous system medications** | Central nervous system | Antidepressants | Paroxetine |
| **Central nervous system medications** | Central nervous system | Antidepressants | Protriptyline |
| **Central nervous system medications** | Central nervous system | Antidepressants | Trimipramine |
| **Central nervous system medications** | Central nervous system | Barbiturates | Amobarbital |
| **Central nervous system medications** | Central nervous system | Barbiturates | Butabarbital |
| **Central nervous system medications** | Central nervous system | Barbiturates | Butalbital |
| **Central nervous system medications** | Central nervous system | Barbiturates | Mephobarbital |
| **Central nervous system medications** | Central nervous system | Barbiturates | Pentobarbital |
| **Central nervous system medications** | Central nervous system | Barbiturates | Phenobarbital |
| **Central nervous system medications** | Central nervous system | Barbiturates | Secobarbital |
| **Central nervous system medications** | Central nervous system | Benzodiazepines | Alprazolam |
| **Central nervous system medications** | Central nervous system | Benzodiazepines | Chlordiazepoxide |
| **Central nervous system medications** | Central nervous system | Benzodiazepines | Clonazepam |
| **Central nervous system medications** | Central nervous system | Benzodiazepines | Clorazepate |
| **Central nervous system medications** | Central nervous system | Benzodiazepines | Diazepam |
| **Central nervous system medications** | Central nervous system | Benzodiazepines | Estazolam |
| **Central nervous system medications** | Central nervous system | Benzodiazepines | Flurazepam |
| **Central nervous system medications** | Central nervous system | Benzodiazepines | Lorazepam |
| **Central nervous system medications** | Central nervous system | Benzodiazepines | Oxazepam |
| **Central nervous system medications** | Central nervous system | Benzodiazepines | Quazepam |
| **Central nervous system medications** | Central nervous system | Benzodiazepines | Temazepam |
| **Central nervous system medications** | Central nervous system | Benzodiazepines | Triazolam |
| **Central nervous system medications** | Central nervous system | z-drugs | Eszopiclone |
| **Central nervous system medications** | Central nervous system | z-drugs | Zaleplon |
| **Central nervous system medications** | Central nervous system | z-drugs | Zolpidem |
| **Endocrine medications** | Endocrine | *not specified* | Estrogens |
| **Endocrine medications** | Endocrine | Long-acting sulfonylureas | Chlorpropamide |
| **Endocrine medications** | Endocrine | Long-acting sulfonylureas | Glimepiride |
| **Endocrine medications** | Endocrine | Long-acting sulfonylureas | Glyburide |
| **Gastrointestinal medications** | Gastrointestinal | *not specified* | Metoclopramide |
| **Gastrointestinal medications** | Gastrointestinal | Proton-pump inhibitors | Lansoprazole |
| **Gastrointestinal medications** | Gastrointestinal | Proton-pump inhibitors | Dexlansoprazole |
| **Gastrointestinal medications** | Gastrointestinal | Proton-pump inhibitors | Omeprazole |
| **Gastrointestinal medications** | Gastrointestinal | Proton-pump inhibitors | Esomeprazole |
| **Gastrointestinal medications** | Gastrointestinal | Proton-pump inhibitors | Pantoprazole |
| **Gastrointestinal medications** | Gastrointestinal | Proton-pump inhibitors | Rabeprazole |
| **Pain medications** | Pain medications | Non-cyclooxygenase-selective  nonsteroidal anti-inflammatory drug (NSAIDs) | Diclofenac |
| **Pain medications** | Pain medications | NSAIDs | Diflunisal |
| **Pain medications** | Pain medications | NSAIDs | Etodolac |
| **Pain medications** | Pain medications | NSAIDs | Fenoprofen |
| **Pain medications** | Pain medications | NSAIDs | Meclofenamate |
| **Pain medications** | Pain medications | NSAIDs | Mefenamic acid |
| **Pain medications** | Pain medications | NSAIDs | Meloxicam |
| **Pain medications** | Pain medications | NSAIDs | Nabumetone |
| **Pain medications** | Pain medications | NSAIDs | Oxaprozin |
| **Pain medications** | Pain medications | NSAIDs | Piroxicam |
| **Pain medications** | Pain medications | NSAIDs | Sulindac |
| **Pain medications** | Pain medications | NSAIDs | Tolmetin |
| **Pain medications** | Pain medications | Other pain medications | Indomethacin |
| **Pain medications** | Pain medications | Other pain medications | Ketorolac |
| **Pain medications** | Pain medications | Other pain medications | Meperidine |
| **Pain medications** | Pain medications | Skeletal muscle relaxants | Carisoprodol |
| **Pain medications** | Pain medications | Skeletal muscle relaxants | Chlorzoxazone |
| **Pain medications** | Pain medications | Skeletal muscle relaxants | Cyclobenzaprine |
| **Pain medications** | Pain medications | Skeletal muscle relaxants | Metaxalone |
| **Pain medications** | Pain medications | Skeletal muscle relaxants | Methocarbamol |
| **Pain medications** | Pain medications | Skeletal muscle relaxants | Orphenadrine |
| **Pain medications** | Pain medications | Skeletal muscle relaxants | Tizanidine |

# Table S2. Censoring events, by age, gender, ethnicity, and race category

| n=802,475* |  | | Death | Disenrollment | Specific date reached | Medication discontinuation |  |
| --- | --- | --- | --- | --- | --- | --- | --- |
| **All older adults** | |  | 101,789 (12.7%) | 173,898 (21.7%) | 353,597 (44.1%) | 173,191 (21.6%) |  |
| **Age category** | 65 to 74 years; n (%) | | 32,313  (7.0%) | 110,135 (23.7%) | 213,302 (45.9%) | 109,448 (23.5%) |  |
|  | ≥75 years; n (%) | | 69,476 (20.6%) | 63,753  (18.9%) | 140,295 (41.6%) | 63,743  (18.9%) |  |
| **Gender** | Female; n (%) | | 55,909  (11.7%) | 106,172 (22.2%) | 208,544 (43.7%) | 107,130 (22.4%) |  |
|  | Male; n (%) | | 45,880 (14.1%) | 67,726  (20.9%) | 145,053 (44.7%) | 66,061  (20.3%) |  |
| **Race** | Asian; n (%) | | 1,761  (12.6%) | 3,088  (22.0%) | 6,061  (43.2%) | 3,123  (22.3%) |  |
|  | Black; n (%) | | 5,604  (13.1%) | 9,936  (23.2%) | 17,877 (41.7%) | 9,419  (22.0%) |  |
|  | No database-recorded race; n (%) | | 63,204 (12.5%) | 108,285 (21.4%) | 226,151 (44.6%) | 109,577 (21.6%) |  |
|  | White; n (%) | | 31,220 (13.1%) | 52,589  (22.1%) | 103,508 (43.4%) | 51,072  (21.4%) |  |
| **Ethnicity** | | Hispanic; n (%) | | 5,609  (12.5%) | 9,913  (22.1%) | 19,288 (42.9%) | 10,139  (22.6%) |
|  | | Non-Hispanic; n (%) | | 28,993 (13.3%) | 48,831  (22.4%) | 93,607 (42.9%) | 46,662  (21.4%) |
|  | | No database-recorded ethnicity, n (%) | | 67,187 (12.5%) | 115,154 (21.4%) | 240,702 (44.6%) | 116,309 (21.6%) |
| *802,475 observations from 729,705 unique individuals. | | | | | | |  |

# Figure S1. Cohort definition flow chart

| **Complete dataset:**  For each of the 16 cohort definitions there were initially 93159383 older adults in the dataset | |  |  |  | | |
| --- | --- | --- | --- | --- | --- | --- |
|  |  |  |  |  | | |
|  |  |  |  |  | | |
|  |  |  | **Step 1 - Excluded due to not being dispensed high-risk medication: 1,458,442,616**   - Antispasmodics: 92922171 - Antihistamines: 88690758 - Antimuscarinics: 92235662 - Antithrombotics: 93143335 - Peripheral alpha blockers: 92727157 - Antidepressants: 91752186 - Barbiturates: 92778869 - Benzodiazepines: 88435945 | - z-drugs: 92038604 - Estrogens: 92890885 - Sulfonylureas: 92542112 - Metoclopramide: 92776208 - PPI: 86940396 - NSAIDs: 88267034 - Other pain medications: 92008862 - Skeletal muscle relaxants: 88292432 | | |
|  |  |  |  |  |  |  |
|  |  |  |  |  | | |
| **Cohort after Step 1: 32,107,512**   - Antispasmodics: 237212 - Antihistamines: 4468625 - Antimuscarinics: 923721 - Antithrombotics: 16048 - Peripheral alpha blockers: 432226 - Antidepressants: 1407197 - Barbiturates: 380514 - Benzodiazepines: 4723438 | - z-drugs: 1120779 - Estrogens: 268498 - Sulfonylureas: 617271 - Metoclopramide: 383175 - PPI: 6218987 - NSAIDs: 4892349 - Other pain medications: 1150521 - Skeletal muscle relaxants: 4866951 |  |  |  | | |
|  |  |  |  |  | | |
|  |  |  |  | | | |
|  |  |  | **Step 2 – Excluded due to insufficient enrolment: 9,615,441**   - Antispasmodics: 67041 - Antihistamines: 1416618 - Antimuscarinics: 246379 - Antithrombotics: 3853 - Peripheral alpha blockers: 119220 - Antidepressants: 445159 - Barbiturates: 132221 - Benzodiazepines: 1420139 | - z-drugs: 358085 - Estrogens: 73582 - Sulfonylureas: 185907 - Metoclopramide: 114309 - PPI: 1783210 - NSAIDs: 1390371 - Other pain medications: 332286 - Skeletal muscle relaxants: 1527061 | | |
|  |  |  |  |  |  |  |
|  |  |  |  |  | | |
| **Cohort after Step 2: 22,492,071**   - Antispasmodics: 170171 - Antihistamines: 3052007 - Antimuscarinics: 677342 - Antithrombotics: 12195 - Peripheral alpha blockers: 313006 - Antidepressants: 962038 - Barbiturates: 248293 - Benzodiazepines: 3303299 | - z-drugs: 762694 - Estrogens: 194916 - Sulfonylureas: 431364 - Metoclopramide: 268866 - PPI: 4435777 - NSAIDs: 3501978 - Other pain medications: 818235 - Skeletal muscle relaxants: 3339890 |  |  |  | | |
|  |  |  |  |  | | |
|  |  |  |  |  | | |
|  |  |  | **Step 3 – Excluded due to less than 2 fills of high-risk medications in the 180 days prior to the index date: 15,303,250**   - Antispasmodics: 149714 - Antihistamines: 2641041 - Antimuscarinics: 384595 - Antithrombotics: 5123 - Peripheral alpha blockers: 146494 - Antidepressants: 473615 - Barbiturates: 195736 - Benzodiazepines: 2137086 | - z-drugs: 389781 - Estrogens: 143897 - Sulfonylureas: 189995 - Metoclopramide: 0 - PPI: 2412672 - NSAIDs: 2588039 - Other pain medications: 751286 - Skeletal muscle relaxants: 2694176 | | |
|  |  |  |  |  |  |  |
|  |  |  |  |  | | |
| **Cohort after Step 3: 7,188,821**   - Antispasmodics: 20457 - Antihistamines: 410966 - Antimuscarinics: 292747 - Antithrombotics: 7072 - Peripheral alpha blockers: 166512 - Antidepressants: 488423 - Barbiturates: 52557 - Benzodiazepines: 1166213 | - z-drugs: 372913 - Estrogens: 51019 - Sulfonylureas: 241369 - Metoclopramide: 268866 - PPI: 2023105 - NSAIDs: 913939 - Other pain medications: 66949 - Skeletal muscle relaxants: 645714 |  |  |  | | |
|  |  |  |  |  | | |
|  |  |  |  | | | |
|  |  |  | **Step 4 – Excluded due to less than 90 days’ supply of high-risk medication in the 180 days prior to the index date: 2,198,084**   - Antispasmodics: 12918 - Antihistamines: 242986 - Antimuscarinics: 43687 - Antithrombotics: 695 - Peripheral alpha blockers: 17669 - Antidepressants: 86470 - Barbiturates: 31272 - Benzodiazepines: 391373 | - z-drugs: 85254 - Estrogens: 12279 - Sulfonylureas: 18369 - Metoclopramide: 242507 - PPI: 278362 - NSAIDs: 353890 - Other pain medications: 52539 - Skeletal muscle relaxants: 327814 | | |
|  |  |  |  |  |  |  |
|  |  |  |  |  | | |
| **Cohort after Step 4: 4,990,737**   - Antispasmodics: 7539 - Antihistamines: 167980 - Antimuscarinics: 249060 - Antithrombotics: 6377 - Peripheral alpha blockers: 148843 - Antidepressants: 401953 - Barbiturates: 21285 - Benzodiazepines: 774840 | - z-drugs: 287659 - Estrogens: 38740 - Sulfonylureas: 223000 - Metoclopramide: 26359 - PPI: 1744743 - NSAIDs: 560049 - Other pain medications: 14410 - Skeletal muscle relaxants: 317900 |  |  |  | | |
|  |  |  |  |  | | |
|  |  |  |  |  | | |
|  |  |  | **Step 5 – Excluded due to prior use of high-risk medication: 4,047,892**   - Antispasmodics: 5984 - Antihistamines: 132997 - Antimuscarinics: 163609 - Antithrombotics: 5249 - Peripheral alpha blockers: 109700 - Antidepressants: 324098 - Barbiturates: 19906 - Benzodiazepines: 713353 | - z-drugs: 257633 - Estrogens: 32856 - Sulfonylureas: 175523 - Metoclopramide: 19341 - PPI: 1387715 - NSAIDs: 418766 - Other pain medications: 9423 - Skeletal muscle relaxants: 271739 | | |
|  |  |  |  |  |  |  |
|  |  |  |  |  | | |
| **Cohort after Step 5: 942,845**   - Antispasmodics: 1555 - Antihistamines: 34983 - Antimuscarinics: 85451 - Antithrombotics: 1128 - Peripheral alpha blockers: 39143 - Antidepressants: 77855 - Barbiturates: 1379 - Benzodiazepines: 61487 | - z-drugs: 30026 - Estrogens: 5884 - Sulfonylureas: 47477 - Metoclopramide: 7018 - PPI: 357028 - NSAIDs: 141283 - Other pain medications: 4987 - Skeletal muscle relaxants: 46161 |  |  |  | | |
|  |  |  |  |  | | |
|  |  |  |  | | | |
|  |  |  | **Step 6 – Excluded due to age younger than 65 years: 139,919**   - Antispasmodics: 626 - Antihistamines: 9486 - Antimuscarinics: 5669 - Antithrombotics: 36 - Peripheral alpha blockers: 4348 - Antidepressants: 20864 - Barbiturates: 353 - Benzodiazepines: 8893 | - z-drugs: 6935 - Estrogens: 1845 - Sulfonylureas: 4160 - Metoclopramide: 1190 - PPI: 43900 - NSAIDs: 22949 - Other pain medications: 786 - Skeletal muscle relaxants: 7879 | | |
|  |  |  |  |  |  |  |
|  |  |  |  |  | | |
| **Cohort after Step 6: 802,926**   - Antispasmodics: 929 - Antihistamines: 25497 - Antimuscarinics: 79782 - Antithrombotics: 1092 - Peripheral alpha blockers: 34795 - Antidepressants: 56991 - Barbiturates: 1026 - Benzodiazepines: 52594 | - z-drugs: 23091 - Estrogens: 4039 - Sulfonylureas: 43317 - Metoclopramide: 5828 - PPI: 313128 - NSAIDs: 118334 - Other pain medications: 4201 - Skeletal muscle relaxants: 38282 |  |  |  | | |
|  |  |  |  |  | | |
|  |  |  |  |  | | |
|  |  |  | **Step 7 – Excluded due to missing age or missing gender: 10**   - Antispasmodics: 0 - Antihistamines: 0 - Antimuscarinics: 0 - Antithrombotics: 0 - Peripheral alpha blockers: 0 - Antidepressants: 1 - Barbiturates: 0 - Benzodiazepines: 1 | - z-drugs: 0 - Estrogens: 0 - Sulfonylureas: 2 - Metoclopramide: 0 - PPI: 4 - NSAIDs: 2 - Other pain medications: 0 - Skeletal muscle relaxants: 0 | | |
|  |  |  |  |  |  |  |
|  |  |  |  |  | | |
| **Cohort after Step 7: 802,916**   - Antispasmodics: 929 - Antihistamines: 25497 - Antimuscarinics: 79782 - Antithrombotics: 1092 - Peripheral alpha blockers: 34795 - Antidepressants: 56990 - Barbiturates: 1026 - Benzodiazepines: 52593 | - z-drugs: 23091 - Estrogens: 4039 - Sulfonylureas: 43315 - Metoclopramide: 5828 - PPI: 313124 - NSAIDs: 118332 - Other pain medications: 4201 - Skeletal muscle relaxants: 38282 |  |  |  | | |
|  |  |  |  |  | | |
|  |  |  |  | | | |
|  |  |  | **Step 8 – Excluded because older adults did not start follow-up period*: 441**   - Antispasmodics: 0 - Antihistamines: 23 - Antimuscarinics: 50 - Antithrombotics: 0 - Peripheral alpha blockers: 19 - Antidepressants: 31 - Barbiturates: 1 - Benzodiazepines: 25 | - z-drugs: 8 - Estrogens: 4 - Sulfonylureas: 17 - Metoclopramide: 3 - PPI: 166 - NSAIDs: 68 - Other pain medications: 1 - Skeletal muscle relaxants: 25 | | |
|  |  |  |  |  |  |  |
|  |  |  |  |  | | |
| **After Step 10 – Final cohort (grouped per-medication class): 802,475**  **Anticholinergics**   - Antispasmodics: 929 - Antihistamines: 25474 - Antimuscarinics: 79732   **Cardiovascular medications:**   - Antithrombotics: 1092 - Peripheral alpha blockers: 34776   **Central nervous system medications:**   - Antidepressants: 56959 - Barbiturates: 1025 - Benzodiazepines: 52568 - z-drugs: 23083   **Endocrine medications:**   - Estrogens: 4035 - Sulfonylureas: 43298   **Gastrointestinal medications:**   - Metoclopramide: 5825 - PPI: 312958   **Pain medications:**   - NSAIDs: 118264 - Other pain medications: 4200 - Skeletal muscle relaxants: 38257 | |  |  |  | | |
|  |  |  |  |  |  |  |

*Individuals not starting the follow-up period: incomplete cases (e.g., missing covariates) or censored before start of the follow-up period.

# Table S3. Continuation of baseline characteristics

| **Characteristics** |  | **Age, gender, ethnicity, and race; n (%)** | | | | | | | | | | |
| --- | --- | --- | --- | --- | --- | --- | --- | --- | --- | --- | --- | --- |
|  | **All older adults** | **Age** | | **Gender** | | **Race** | | | | **Ethnicity** | | |
|  |  | **65-74** | **≥75** | **Female** | **Male** | **Asian** | **Black** | **No database-recorded race** | **White** | **Hispanic** | **Non-Hispanic** | **No database recorded-ethnicity** |
|  | n = 729,705* | n = 424,299 | n = 305,406 | n = 432,140 | n = 297,565 | n = 12,777 | n = 39,020 | n = 460,801 | n = 217,107 | n = 40,977 | n = 198,621 | n = 490,107 |
| **Comorbidities, n (%)** | | | | | | | | | |  |  |  |
| Hypertension | 592976 (81.3%) | 329240 (77.6%) | 263736 (86.4%) | 344775 (79.8%) | 248201 (83.4%) | 10350 (81.0%) | 32616 (83.6%) | 373698 (81.1%) | 176305 (81.2%) | 33413 (81.5%) | 162259 (81.7%) | 397299 (81.1%) |
| Complicated diabetes | 211175 (28.9%) | 119810 (28.2%) | 91365 (29.9%) | 114350 (26.5%) | 96825 (32.5%) | 3881 (30.4%) | 12419 (31.8%) | 133924 (29.1%) | 60964 (28.1%) | 12848 (31.4%) | 56946 (28.7%) | 141397 (28.9%) |
| Cardiac arrhythmias | 209816 (28.8%) | 94685 (22.3%) | 115131 (37.7%) | 110795 (25.6%) | 99021 (33.3%) | 3592 (28.1%) | 11202 (28.7%) | 130753 (28.4%) | 64256 (29.6%) | 11527 (28.1%) | 59266 (29.8%) | 139009 (28.4%) |
| Chronic pulmonary disease | 194899 (26.7%) | 109944 (25.9%) | 84955 (27.8%) | 118731 (27.5%) | 76168 (25.6%) | 3301 (25.8%) | 10677 (27.4%) | 122398 (26.6%) | 58503 (26.9%) | 10633 (25.9%) | 53866 (27.1%) | 130390 (26.6%) |
| Renal failure | 144202 (19.8%) | 62877 (14.8%) | 81325 (26.6%) | 78953 (18.3%) | 65249 (21.9%) | 2646 (20.7%) | 8625 (22.1%) | 90577 (19.7%) | 42357 (19.5%) | 8351 (20.4%) | 39884 (20.1%) | 95959 (19.6%) |
| Peripheral vascular disorder | 190754 (26.1%) | 86949 (20.5%) | 103805 (34.0%) | 103195 (23.9%) | 87559 (29.4%) | 3360 (26.3%) | 10459 (26.8%) | 121018 (26.3%) | 55962 (25.8%) | 10922 (26.7%) | 52029 (26.2%) | 127844 (26.1%) |
| Congestive heart failure | 126903 (17.4%) | 53708 (12.7%) | 73195 (24.0%) | 69249 (16.0%) | 57654 (19.4%) | 2193 (17.2%) | 7231 (18.5%) | 79297 (17.2%) | 38168 (17.6%) | 7015 (17.1%) | 35639 (17.9%) | 84230 (17.2%) |
| Dementia | 72041 (9.9%) | 16437 (3.9%) | 55604 (18.2%) | 46237 (10.7%) | 25804 (8.7%) | 1253 (9.8%) | 4057 (10.4%) | 45061 (9.8%) | 21667 (10.0%) | 4041 (9.9%) | 20194 (10.2%) | 47803 (9.8%) |
| Weight loss | 66236 (9.1%) | 29519 (7.0%) | 36717 (12.0%) | 38501 (8.9%) | 27735 (9.3%) | 1149 (9.0%) | 3839 (9.8%) | 41553 (9.0%) | 19709 (9.1%) | 3748 (9.1%) | 18372 (9.2%) | 44128 (9.0%) |
| Hemiplegia | 16656 (2.3%) | 8449 (2.0%) | 8207 (2.7%) | 8991 (2.1%) | 7665 (2.6%) | 262 (2.1%) | 1040 (2.7%) | 10510 (2.3%) | 4852 (2.2%) | 909  (2.2%) | 4704 (2.4%) | 11050 (2.3%) |
| Alcohol abuse | 22452 (3.1%) | 15813 (3.7%) | 6639 (2.2%) | 7851 (1.8%) | 14601 (4.9%) | 386 (3.0%) | 1194 (3.1%) | 14391 (3.1%) | 6477 (3.0%) | 1232 (3.0%) | 6019 (3.0%) | 15202 (3.1%) |
| Any tumor | 104803 (14.4%) | 54169 (12.8%) | 50634 (16.6%) | 47128 (10.9%) | 57675 (19.4%) | 1847 (14.5%) | 5918 (15.2%) | 65249 (14.2%) | 31797 (14.6%) | 5771 (14.1%) | 29342 (14.8%) | 69698 (14.2%) |
| Metastatic cancer | 19642 (2.7%) | 11103 (2.6%) | 8539 (2.8%) | 9739 (2.3%) | 9903 (3.3%) | 360 (2.8%) | 1100 (2.8%) | 12185 (2.6%) | 5997 (2.8%) | 1067 (2.6%) | 5458 (2.7%) | 13125 (2.7%) |
| Coagulopathy | 51775 (7.1%) | 24938 (5.9%) | 26837 (8.8%) | 24525 (5.7%) | 27250 (9.2%) | 922 (7.2%) | 2738 (7.0%) | 32621 (7.1%) | 15492 (7.1%) | 3022 (7.4%) | 14212 (7.2%) | 34544 (7.0%) |
| Deficiency anemias | 98799 (13.5%) | 47795 (11.3%) | 51004 (16.7%) | 59827 (13.8%) | 38972 (13.1%) | 1669 (13.1%) | 5751 (14.7%) | 61687 (13.4%) | 29711 (13.7%) | 5568 (13.6%) | 27453 (13.8%) | 65810 (13.4%) |
| Fluid and electrolyte disorders | 158561 (21.7%) | 75410 (17.8%) | 83151 (27.2%) | 94847 (21.9%) | 63714 (21.4%) | 2734 (21.4%) | 9038 (23.2%) | 99066 (21.5%) | 47761 (22.0%) | 8903 (21.7%) | 44618 (22.5%) | 105061 (21.4%) |
| Liver disease | 66857 (9.2%) | 44606 (10.5%) | 22251 (7.3%) | 37194 (8.6%) | 29663 (10.0%) | 1196 (9.4%) | 3595 (9.2%) | 42165 (9.2%) | 19912 (9.2%) | 3955 (9.7%) | 17926 (9.0%) | 44993 (9.2%) |
| Psychosis | 14761 (2.0%) | 7661 (1.8%) | 7100 (2.3%) | 9491 (2.2%) | 5270 (1.8%) | 267 (2.1%) | 890 (2.3%) | 9163 (2.0%) | 4438 (2.0%) | 851  (2.1%) | 4152 (2.1%) | 9760 (2.0%) |
| Pulmonary circulation disorders | 23629 (3.2%) | 11054 (2.6%) | 12575 (4.1%) | 13925 (3.2%) | 9704 (3.3%) | 417 (3.3%) | 1438 (3.7%) | 14533 (3.2%) | 7238 (3.3%) | 1290 (3.1%) | 6803 (3.4%) | 15535 (3.2%) |
| HIV/AIDS | 1647 (0.2%) | 1355 (0.3%) | 292 (0.1%) | 493 (0.1%) | 1154 (0.4%) | 32 (0.3%) | 140 (0.4%) | 1044 (0.2%) | 431 (0.2%) | 93 (0.2%) | 457 (0.2%) | 1096 (0.2%) |
| **Year of cohort entry** | | | | | | | | | | | | |
| 2017; n (%) | 90179 (12.4%) | 52441 (12.4%) | 37738 (12.4%) | 53700 (12.4%) | 36479 (12.3%) | 1671 (13.1%) | 5113 (13.1%) | 55848 (12.1%) | 27547 (12.7%) | 5276 (12.9%) | 25325 (12.8%) | 59578 (12.2%) |
| 2018; n (%) | 91760 (12.6%) | 53454 (12.6%) | 38306 (12.5%) | 54262 (12.6%) | 37498 (12.6%) | 1677 (13.1%) | 5145 (13.2%) | 56865 (12.3%) | 28073 (12.9%) | 5246 (12.8%) | 26110 (13.1%) | 60404 (12.3%) |
| 2019; n (%) | 94769 (13.0%) | 55961 (13.2%) | 38808 (12.7%) | 55900 (12.9%) | 38869 (13.1%) | 1694 (13.3%) | 5345 (13.7%) | 58974 (12.8%) | 28756 (13.2%) | 5457 (13.3%) | 26314 (13.2%) | 62998 (12.9%) |
| 2020; n (%) | 97720 (13.4%) | 57928 (13.7%) | 39792 (13.0%) | 57846 (13.4%) | 39874 (13.4%) | 1720 (13.5%) | 5354 (13.7%) | 61395 (13.3%) | 29251 (13.5%) | 5746 (14.0%) | 26703 (13.4%) | 65271 (13.3%) |
| 2021; n (%) | 119958 (16.4%) | 64947 (15.3%) | 55011 (18.0%) | 71773 (16.6%) | 48185 (16.2%) | 2055 (16.1%) | 6124 (15.7%) | 76832 (16.7%) | 34947 (16.1%) | 6439 (15.7%) | 31867 (16.0%) | 81652 (16.7%) |
| 2022; n (%) | 113866 (15.6%) | 67884 (16.0%) | 45982 (15.1%) | 67125 (15.5%) | 46741 (15.7%) | 1944 (15.2%) | 5944 (15.2%) | 71902 (15.6%) | 34076 (15.7%) | 6372 (15.6%) | 31265 (15.7%) | 76229 (15.6%) |
| 2023, n (%) | 121453 (16.6%) | 71684 (16.9%) | 49769 (16.3%) | 71534 (16.6%) | 49919 (16.8%) | 2016 (15.8%) | 5995 (15.4%) | 78985 (17.1%) | 34457 (15.9%) | 6441 (15.7%) | 31037 (15.6%) | 83975 (17.1%) |

*This table is restricted to older adults’ first cohort entry. Our dataset consists of 802,475 observations from 729,705 unique individuals.

# Table S4. Discontinuation vs. continuation by medication type

|  |  | **Number of older adults sorted by whether they discontinued their high-risk medication use (n=802,475)*** | |
| --- | --- | --- | --- |
|  |  | Continuation | Discontinuation |
| All older adults | n | 621,363 (77.4%) | 181,112 (22.6%) |
| **By high-risk medication type** | | | |
| Anticholinergic | 106,135 | 84,262 (79.4%) | 21,873 (20.6%) |
| Central nervous system | 133,635 | 106,753 (79.9%) | 26,882 (20.1%) |
| Endocrine | 47,333 | 38,117 (80.5%) | 9,216 (19.5%) |
| Gastrointestinal | 318,783 | 248,155 (77.8%) | 70,628 (22.2%) |
| Pain medications | 160,721 | 113,462 (70.6%) | 47,259 (29.4%) |
| Cardiovascular | 35,868 | 30,614 (85.4%) | 5,254 (14.7%) |
| *802,475 observations from 729,705 unique individuals. Among these unique individuals, 166,613 (22.8%) discontinued at least one high-risk medication. | | | |

# Table S5. Complete results for the association of age, gender, ethnicity, and race and the discontinuation of high-risk medications (Cox regression model)

| **Variable** | **Adjusted model without interaction terms**  (n=802,475) | **Adjusted model with interaction terms^1^**  (n=802,475) |
| --- | --- | --- |
|  |  | *Hazard ratio* (95% CI) |
| **Age category** (ref. 65 to 74 years) |  |  |
| ≥75 years | **0.88 (0.87 to 0.89)***** | **0.86 (0.84 to 0.88)***** |
| **Gender** (ref. Female) |  |  |
| Male | **0.91 (0.90 to 0.92)***** | **0.89 (0.87 to 0.91)***** |
| **Race** (ref. White) |  |  |
| Asian | 1.05 (1.02 to 1.09)** | 1.06 (0.99 to 1.13) |
| Black | **1.04 (1.01 to 1.06)***** | **1.07 (1.03 to 1.11)**** |
| No database-recorded race | 1.02 (1.00 to 1.03)* | 1.03 (1.01 to 1.06)* |
| **Ethnicity** (ref. Non-Hispanic) |  |  |
| Hispanic | **1.05 (1.02 to 1.07)***** | 1.02 (0.97 to 1.07) |
| No database-recorded ethnicity | 1.01 (1.00 to 1.02) | 1.03 (1.00 to 1.05)* |
| **Interaction terms** |  |  |
| *Race # Ethnicity* |  |  |
| Black # Hispanic | - | 0.96 (0.83 to 1.12) |
| Black # No database-recorded ethnicity | - | 0.94 (0.90 to 0.98)** |
| Asian # Hispanic | - | 1.07 (0.89 to 1.29) |
| Asian # No database-recorded ethnicity | - | 0.96 (0.90 to 1.04) |
| No database-recorded race # Hispanic | - | 1.01 (0.97 to 1.06) |
| No database-recorded race # No database-recorded ethnicity | - | 0.96 (0.94 to 0.99)** |
| *Race # Gender* |  |  |
| Black # Male | - | (0.97 to 1.06) |
| Asian # Male | - | (0.97 to 1.06) |
| No database-recorded race # Male | - | (0.97 to 1.06) |
| *Race # Age category* |  |  |
| Black # ≥75 years | - | 1.00 (0.96 to 1.05) |
| Asian # ≥75 years | - | 0.99 (0.93 to 1.07) |
| No database-recorded # ≥75 years |  | 0.99 (0.97 to 1.02) |
| *Ethnicity # Gender* |  |  |
| Hispanic # Male | - | 1.00 (0.96 to 1.05) |
| No database-recorded ethnicity # Male | - | 1.01 (0.98 to 1.03) |
| *Ethnicity # Age category* |  |  |
| Hispanic # ≥75 years | - | 1.02 (0.97 to 1.06) |
| No database-recorded ethnicity # ≥75 years | - | 1.01 (0.99 to 1.04) |
| *Gender # Age category* |  |  |
| Male # ≥75 years | - | **1.04 (1.02 to 1.06)***** |
| Insurance type (ref. Commercial insurance) |  |  |
| Medicare Advantage | **1.22 (1.19 to 1.25)***** | **1.22 (1.19 to 1.25)***** |
| Copay for all medications during the baseline period | **1.00 (1.00 to 1.00)***** | **1.00 (1.00 to 1.00)***** |
| Copay for high-risk medication at index date | 1.00 (1.00 to 1.00) | 1.00 (1.00 to 1.00) |
| Combined Comorbidity Score (per 1-unit increase) | 1.00 (0.99 to 1.00) | 1.00 (0.99 to 1.00) |
| Comorbidities |  |  |
| Congestive heart failure | **0.95 (0.92 to 0.97)***** | **0.95 (0.92 to 0.97)***** |
| Dementia | **0.73 (0.71 to 0.76)***** | **0.73 (0.71 to 0.76)***** |
| Renal failure | **0.95 (0.93 to 0.97)***** | **0.95 (0.93 to 0.97)***** |
| Hemiplegia | **0.91 (0.88 to 0.95)***** | **0.91 (0.88 to 0.95)***** |
| Alcohol abuse | **1.06 (1.03 to 1.09)***** | **1.06 (1.03 to 1.10)***** |
| Any tumor | 0.95 (0.89 to 1.01) | 0.95 (0.89 to 1.01) |
| Metastatic cancer | 0.98 (0.97 to 1) | 0.98 (0.96 to 1.00)* |
| Cardiac arrhythmias | **0.96 (0.94 to 0.97)***** | **0.96 (0.94 to 0.97)***** |
| Chronic pulmonary disease | 1.02 (1 to 1.03)* | 1.02 (1.00 to 1.03)* |
| Coagulopathy | 0.98 (0.96 to 1) | 0.98 (0.95 to 1.00)* |
| Deficiency anemias | 1.01 (1 to 1.03) | 1.01 (1.00 to 1.03) |
| Fluid and electrolyte disorders | **0.95 (0.93 to 0.97)***** | **0.95 (0.93 to 0.97)***** |
| Liver disease | **1.08 (1.06 to 1.10)***** | **1.08 (1.06 to 1.10)***** |
| Peripheral vascular disorder | 1 (0.98 to 1.01) | 1.00 (0.98 to 1.01) |
| Psychosis | **0.86 (0.82 to 0.90)***** | **0.86 (0.82 to 0.90)***** |
| Pulmonary circulation disorders | 0.96 (0.93 to 0.99)** | 0.96 (0.93 to 0.99)** |
| HIV/AIDS | 1.13 (1.02 to 1.25)* | 1.13 (1.02 to 1.25)* |
| Hypertension | **0.95 (0.94 to 0.97)***** | **0.95 (0.94 to 0.97)***** |
| Diabetes | **0.94 (0.93 to 0.96)***** | **0.94 (0.93 to 0.96)***** |
| Number of medications (per 1-unit increase) | **1.01 (1.01 to 1.01)***** | **1.01 (1.01 to 1.01)***** |
| Number of days hospitalized (per 1-unit increase) | 1.00 (1.00 to 1.00)** | 1.00 (1.00 to 1.00)** |
| Number of outpatient visits (per 1-unit increase) | 1.00 (1.00 to 1.00)* | 1.00 (1.00 to 1.00)* |
| Frailty score | 0.98 (0.97 to 1.00)** | 0.98 (0.97 to 1.00)* |
| Cohort entry year |  |  |
| 2018 | **0.96 (0.94 to 0.98)***** | **0.96 (0.94 to 0.98)***** |
| 2019 | **0.91 (0.9 to 0.93)***** | **0.91 (0.90 to 0.93)***** |
| 2020 | **0.86 (0.84 to 0.87)***** | **0.86 (0.84 to 0.87)***** |
| 2021 | **0.77 (0.76 to 0.79)***** | **0.77 (0.76 to 0.79)***** |
| 2022 | **0.71 (0.7 to 0.72)***** | **0.71 (0.70 to 0.72)***** |
| 2023 | **0.71 (0.69 to 0.73)***** | **0.71 (0.69 to 0.73)***** |
| ^1^ Adjusted for the following baseline characteristics: type of healthcare insurance, copay for medications during the baseline period, copay for the high-risk medication at the index date, the Combined Comorbidity Score, comorbidities (as measured for Combined Comorbidity Score: congestive heart failure, dementia, renal failure, weight loss, hemiplegia, alcohol abuse, any tumor, metastatic cancer, cardiac arrhythmias, chronic pulmonary disease, coagulopathy, complicated diabetes, deficiency anemias, fluid and electrolyte disorders, liver disease, peripheral vascular disorder, psychosis, pulmonary circulation disorders, HIV/AIDS, hypertension), number of medications filled, number of days hospitalized, number of outpatient visits, claims-based frailty index, and the year of cohort entry. │ Non-Bonferroni-adjusted p-values: * p<0.05, ** p<0.01, *** p<0.001 - Bonferroni adjusted p-values considered statistically significant are highlighted in **bold** text: ≤0.00208333 (0.05/24 comparisons) │ Adjusted for clustering at the patient level. | | |

# Table S6. Association of age, gender, ethnicity, and race and the discontinuation of high-risk medications (Cox regression model): Analyses without interaction terms, subgroup and sensitivity analyses

| **Variables** | **All medication categories** | **Main model without interaction terms** | **By polypharmacy status** | | **Minimum 1 ambulatory visit** *(during the baseline period)* | **Main model restricted to first cohort entry** | **Follow-up period capped at 365 days** | **Accelerated failure time model** | **Analyses restricted to American Geriatric Society Beers Criteria ® ^1^** |
| --- | --- | --- | --- | --- | --- | --- | --- | --- | --- |
|  |  |  | *Fewer than 5 medications* | ≥*5 medications* |  |  |  |  |  |
| **n =** | **802,475** | **802,475** | **40,094** | **762,381** | **794,493** | **729,705** | **355,398** | **802,475** | **788,630** |
| **Race (ref. White)** | HR (95% CI) | HR (95% CI) | HR (95% CI) | HR (95% CI) | HR (95% CI) | HR (95% CI) | HR (95% CI) | HR (95% CI) | HR (95% CI) |
| Asian | 1.06  (0.99 to 1.13) | 1.05**  (1.02 to 1.09) | 1.15  (0.9 to 1.47) | 1.05  (0.98 to 1.12) | 1.06  (1.03 to 1.11) | 1.05  (0.98 to 1.12) | 1.04  (0.96 to 1.12) | 1.06  (0.99 to 1.13) | 1.05  (0.99 to 1.12) |
| Black | **1.07****  (1.03 to 1.11) | **1.04*****  (1.01 to 1.06) | 1.05  (0.89 to 1.23) | **1.07****  (1.03 to 1.11) | **1.07****  (0.99 to 1.13) | **1.07****  (1.03 to 1.11) | 1.00  (0.96 to 1.05) | 1.07***  (1.04 to 1.11) | 1.06**  (1.02 to 1.10) |
| No database-recorded race | 1.03*  (1.01 to 1.06) | 1.02*  (1.00 to 1.03) | 1.03  (0.93 to 1.15) | 1.03  (1.01 to 1.06) | 1.03*  (1.01 to 1.06) | 1.03*  (1 to 1.06) | 1.03*  (1 to 1.07) | 1.03*  (1.01 to 1.06) | 1.03*  (1 to 1.06) |
| **Ethnicity (ref. Non-Hispanic)** |  |  |  |  |  |  |  |  |  |
| Hispanic | 1.02  (0.97 to 1.07) | **1.05*****  (1.02 to 1.07) | 0.91  (0.74 to 1.12) | 1.03  (0.98 to 1.08) | 1.02  (0.98 to 1.07) | 1.03  (0.98 to 1.08) | 1.01  (0.95 to 1.07) | 1.02  (0.98 to 1.07) | 1.02  (0.97 to 1.07) |
| No database-recorded ethnicity | 1.03*  (1.00 to 1.05) | 1.01  (1.00 to 1.02) | 0.98  (0.89 to 1.08) | 1.03**  (1.01 to 1.05) | 1.03*  (1.00 to 1.05) | 1.03*  (1.01 to 1.05) | 1.01  (0.98 to 1.04) | 1.03*  (1 to 1.05) | 1.03*  (1.00 to 1.05) |
| **Gender (ref. Female)** |  |  |  |  |  |  |  |  |  |
| Male | **0.89*****  (0.87 to 0.91) | **0.91*****  (0.90 to 0.92) | **0.87****  (0.79 to 0.95) | **0.89*****  (0.87 to 0.91) | **0.89*****  (0.87 to 0.91) | **0.89*****  (0.87 to 0.91) | **0.92*****  (0.89 to 0.94) | **0.88*****  (0.86 to 0.9) | **0.89*****  (0.87 to 0.91) |
| **Age category (ref. 65-74 years)** |  |  |  |  |  |  |  |  |  |
| 75 and above | **0.86*****  (0.84 to 0.88) | **0.88*****  (0.87 to 0.89) | 0.89*  (0.81 to 0.99) | **0.86*****  (0.84 to 0.88) | **0.86*****  (0.84 to 0.88) | **0.86*****  (0.84 to 0.88) | **0.90*****  (0.87 to 0.92) | **0.87*****  (0.85 to 0.89) | **0.87*****  (0.85 to 0.89) |
| **Interaction terms** |  |  |  |  |  |  |  |  |  |
| *Race # Ethnicity* |  |  |  |  |  |  |  |  |  |
| Black # Hispanic | 0.96  (0.83 to 1.12) | - | 0.81  (0.41 to 1.58) | 0.97  (0.83 to 1.13) | 0.96  (0.82 to 1.11) | 0.99  (0.85 to 1.16) | 1.08  (0.91 to 1.29) | 0.94  (0.81 to 1.10) | 0.96  (0.82 to 1.11) |
| Black # No database-recorded ethnicity | 0.94**  (0.90 to 0.98) | - | 0.94  (0.78 to 1.14) | 0.94**  (0.90 to 0.98) | 0.94**  (0.9 to 0.98) | 0.95*  (0.9 to 0.99) | 1.06  (1 to 1.12) | **0.93****  (0.89 to 0.97) | 0.95*  (0.9 to 0.99) |
| Asian # Hispanic | 1.07  (0.89 to 1.29) | - | 0.84  (0.36 to 1.95) | 1.09  (0.9 to 1.32) | 1.07  (0.89 to 1.29) | 1.06  (0.88 to 1.28) | 1.20  (0.97 to 1.49) | 1.05  (0.87 to 1.25) | 1.05  (0.86 to 1.27) |
| Asian # No database-recorded ethnicity | 0.96  (0.90 to 1.04) | - | 1.08  (0.8 to 1.44) | 0.96  (0.89 to 1.03) | 0.97  (0.9 to 1.04) | 0.96  (0.89 to 1.03) | 0.98  (0.9 to 1.07) | 0.96  (0.89 to 1.03) | 0.96  (0.89 to 1.04) |
| No database-recorded race # Hispanic | 1.01  (0.97 to 1.06) | - | 1.06  (0.86 to 1.31) | 1.01  (0.96 to 1.06) | 1.01  (0.96 to 1.06) | 1.01  (0.96 to 1.07) | 1.02  (0.96 to 1.09) | 1.01  (0.96 to 1.06) | 1.01  (0.96 to 1.06) |
| No database-recorded race # No database-recorded ethnicity | 0.96**  (0.94 to 0.99) | - | 0.94  (0.84 to 1.05) | 0.96**  (0.94 to 0.99) | 0.96**  (0.94 to 0.99) | 0.96**  (0.94 to 0.99) | 0.97  (0.94 to 1.01) | 0.96**  (0.94 to 0.99) | 0.96**  (0.94 to 0.99) |
| *Race # Gender* |  |  |  |  |  |  |  |  |  |
| Black # Male | 0.98  (0.83 to 1.12) | - | 0.96  (0.79 to 1.16) | 0.98  (0.94 to 1.03) | 0.98  (0.94 to 1.03) | 0.98  (0.94 to 1.03) | 1.00  (0.95 to 1.06) | 0.99  (0.94 to 1.03) | 0.98  (0.94 to 1.03) |
| Asian # Male | 1.03  (0.96 to 1.11) | - | 0.98  (0.73 to 1.32) | 1.03  (0.95 to 1.11) | 1.03  (0.96 to 1.11) | 1.05  (0.97 to 1.13) | 1.02  (0.93 to 1.12) | 1.04  (0.96 to 1.11) | 1.04  (0.97 to 1.12) |
| No database-recorded race # Male | 1.01  (0.99 to 1.04) | - | 1.04  (0.94 to 1.15) | 1.01  (0.98 to 1.03) | 1.01  (0.99 to 1.04) | 1.01  (0.99 to 1.04) | 1.02  (0.99 to 1.05) | 1.01  (0.99 to 1.04) | 1.01  (0.99 to 1.04) |
| *Race # Age category* |  |  |  |  |  |  |  |  |  |
| Black # ≥75 years | 1.00  (0.96 to 1.03) | - | 1.06  (0.86 to 1.30) | 1.00  (0.96 to 1.05) | 1.01  (0.97 to 1.06) | 1.00  (0.96 to 1.05) | 0.97  (0.91 to 1.02) | 1.00  (0.96 to 1.05) | 1.02  (0.97 to 1.06) |
| Asian # ≥75 years | 0.99  (0.93 to 1.07) | - | 0.77  (0.54 to 1.09) | 1.01  (0.94 to 1.09) | 1.00  (0.93 to 1.07) | 1.02  (0.94 to 1.10) | 0.97  (0.89 to 1.07) | 0.99  (0.92 to 1.07) | 1.00  (0.93 to 1.07) |
| No database-recorded race # ≥75 years | 0.99  (0.97 to 1.02) | - | 1.01  (0.91 to 1.13) | 1.00  (0.97 to 1.02) | 1.00  (0.97 to 1.02) | 1.00  (0.97 to 1.02) | 0.97  (0.94 to 1.00) | 1.00  (0.97 to 1.02) | 1.00  (0.98 to 1.03) |
| *Ethnicity # Gender* |  |  |  |  |  |  |  |  |  |
| Hispanic # Male | 1.00  (0.96 to 1.05) | - | 1.00  (0.82 to 1.21) | 1.00  (0.96 to 1.05) | 1.00  (0.96 to 1.05) | 0.99  (0.95 to 1.04) | 0.97  (0.92 to 1.03) | 1.00  (0.96 to 1.05) | 1.00  (0.96 to 1.05) |
| No database-recorded ethnicity # Male | 1.01  (0.98 to 1.03) | - | 1.04  (0.93 to 1.15) | 1.01  (0.98 to 1.03) | 1.01  (0.98 to 1.03) | 1.00  (0.98 to 1.03) | 1.00  (0.97 to 1.03) | 1.01  (0.98 to 1.04) | 1.01  (0.98 to 1.04) |
| *Ethnicity # Age category* |  |  |  |  |  |  |  |  |  |
| Hispanic # ≥75 years | 1.02  (0.97 to 1.06) | - | 1.05  (0.85 to 1.29) | 1.01  (0.97 to 1.06) | 1.02  (0.98 to 1.07) | 1.02  (0.97 to 1.07) | 1.02  (0.96 to 1.08) | 1.02  (0.97 to 1.07) | 1.02  (0.97 to 1.06) |
| No database-recorded ethnicity # ≥75 years | 1.01  (0.99 to 1.04) | - | 1.03  (0.92 to 1.15) | 1.01  (0.99 to 1.04) | 1.01  (0.99 to 1.04) | 1.01  (0.99 to 1.04) | 1.03  (1.00 to 1.06) | 1.01  (0.99 to 1.04) | 1.01  (0.99 to 1.04) |
| *Gender # Age category* |  |  |  |  |  |  |  |  |  |
| Male # ≥75 years | **1.04*****  (1.02 to 1.06) | - | 1.03  (0.95 to 1.13) | **1.04*****  (1.02 to 1.06) | **1.04*****  (1.02 to 1.06) | **1.04*****  (1.02 to 1.06) | 1.01  (0.99 to 1.04) | **1.05*****  (1.02 to 1.07) | **1.04****  (1.01 to 1.06) |

| **Variables** | **All medication categories** | **By cohort entry year** | | | | | | | **Chronic high-risk medication use in baseline period defined with 365 day time window ^2^** |
| --- | --- | --- | --- | --- | --- | --- | --- | --- | --- |
|  |  | *2017* | *2018* | *2019* | *2020* | *2021* | *2022* | *2023* |  |
| **n =** | **802,475** | **93,998** | **98,266** | **103,451** | **108,384** | **134,452** | **127,649** | **136,275** | **727,042** |
| **Race (ref. White)** | HR (95% CI) | HR (95% CI) | HR (95% CI) | HR (95% CI) | HR (95% CI) | HR (95% CI) | HR (95% CI) | HR (95% CI) | HR (95% CI) |
| Asian | 1.06  (0.99 to 1.13) | 1.08  (0.93 to 1.26) | 1.02  (0.87 to 1.19) | 1.10  (0.94 to 1.28) | 1.12  (0.95 to 1.32) | 0.98  (0.83 to 1.15) | 1.10  (0.9 to 1.34) | 0.88  (0.62 to 1.24) | 0.98**  (0.91 to 1.07) |
| Black | **1.07****  (1.03 to 1.06) | 1.08  (0.99 to 1.18) | 0.97  (0.89 to 1.06) | 1.02  (0.93 to 1.12) | **1.16****  (1.06 to 1.27) | 1.14**  (1.04 to 1.25) | 1.03  (0.92 to 1.15) | 1.12  (0.92 to 1.36) | 1.07  (1.02 to 1.12) |
| No database-recorded race | 1.03*  (1.01 to 1.06) | 1.02  (0.96 to 1.08) | 1.00  (0.95 to 1.07) | 1.01  (0.95 to 1.07) | 1.07*  (1 to 1.14) | 1.06  (1 to 1.13) | 1.06  (0.98 to 1.14) | 1.01  (0.88 to 1.15) | 1.03  (0.99 to 1.06) |
| **Ethnicity (ref. Non-Hispanic)** |  |  |  |  |  |  |  |  |  |
| Hispanic | 1.02  (0.97 to 1.07) | 0.99  (0.89 to 1.10) | 1.09  (0.98 to 1.21) | 1.10  (0.99 to 1.23) | 0.93  (0.83 to 1.04) | 0.94  (0.83 to 1.06) | 1.12  (0.98 to 1.27) | 0.96  (0.76 to 1.22) | 0.99  (0.94 to 1.06) |
| No database-recorded ethnicity | 1.03*  (1.00 to 1.05) | 0.99  (0.94 to 1.05) | 1.04  (0.99 to 1.10) | 1.06*  (1.01 to 1.12) | 0.99  (0.94 to 1.06) | 1.05  (0.99 to 1.11) | 1.00  (0.93 to 1.07) | 1.06  (0.94 to 1.19) | 1.01  (0.98 to 1.04) |
| **Gender (ref. Female)** |  |  |  |  |  |  |  |  |  |
| Male | **0.89*****  (0.87 to 0.91) | **0.86*****  (0.82 to 0.91) | **0.86*****  (0.81 to 0.9) | **0.87*****  (0.82 to 0.92) | 0.95*  (0.89 to 1.00) | **0.89*****  (0.85 to 0.94) | 0.91**  (0.85 to 0.97) | 0.92  (0.83 to 1.03) | **0.90*****  (0.87 to 0.92) |
| **Age category (ref. 65-74 years)** |  |  |  |  |  |  |  |  |  |
| 75 and above | **0.86*****  (0.84 to 0.88) | **0.82*****  (0.78 to 0.87) | **0.90*****  (0.86 to 0.95) | **0.87*****  (0.82 to 0.91) | **0.85*****  (0.81 to 0.90) | **0.81*****  (0.77 to 0.86) | 0.92*  (0.86 to 0.98) | 1.00  (0.9 to 1.12) | **0.95*****  (0.93 to 0.98) |
| **Interaction terms** |  |  |  |  |  |  |  |  |  |
| *Race # Ethnicity* |  |  |  |  |  |  |  |  |  |
| Black # Hispanic | 0.96  (0.83 to 1.12) | 1.00  (0.71 to 1.41) | 0.83  (0.56 to 1.22) | 1.04  (0.72 to 1.52) | 0.97  (0.65 to 1.45) | 1.29  (0.93 to 1.79) | 0.43**  (0.24 to 0.80) | 1.43  (0.77 to 2.65) | 0.89  (0.73 to 1.08) |
| Black # No database-recorded ethnicity | 0.94**  (0.90 to 0.98) | 0.86**  (0.77 to 0.95) | 1.01  (0.91 to 1.12) | 0.98  (0.89 to 1.09) | 0.94  (0.84 to 1.05) | 0.94  (0.84 to 1.05) | 0.90  (0.79 to 1.03) | 0.97  (0.78 to 1.21) | 0.94*  (0.89 to 1.00) |
| Asian # Hispanic | 1.07  (0.89 to 1.29) | 0.69  (0.43 to 1.10) | 0.86  (0.52 to 1.41) | 1.10  (0.7 to 1.73) | 1.53*  (1.00 to 2.33) | 1.56  (0.99 to 2.46) | 1.24  (0.74 to 2.06) | 0.91  (0.34 to 2.45) | 0.91  (0.72 to 1.15) |
| Asian # No database-recorded ethnicity | 0.96  (0.90 to 1.04) | 0.83*  (0.7 to 0.99) | 0.86  (0.73 to 1.03) | 0.98  (0.82 to 1.17) | 0.94  (0.78 to 1.13) | 1.25*  (1.05 to 1.49) | 0.97  (0.78 to 1.21) | 1.12  (0.78 to 1.61) | 0.96  (0.88 to 1.06) |
| No database-recorded race # Hispanic | 1.01  (0.97 to 1.06) | 0.97  (0.86 to 1.09) | 1.03  (0.92 to 1.16) | 0.98  (0.87 to 1.11) | 1.05  (0.93 to 1.18) | 1.09  (0.96 to 1.23) | 0.94  (0.82 to 1.08) | 1.10  (0.85 to 1.42) | 1.02  (0.96 to 1.08) |
| No database-recorded race # No database-recorded ethnicity | 0.96**  (0.94 to 0.99) | 0.98  (0.92 to 1.04) | 0.97  (0.91 to 1.04) | 0.96  (0.9 to 1.02) | 0.97  (0.91 to 1.04) | 0.91  (0.85 to 0.98) | 0.97  (0.9 to 1.05) | 1.02  (0.89 to 1.17) | 0.97  (0.94 to 1.00) |
| *Race # Gender* |  |  |  |  |  |  |  |  |  |
| Black # Male | 0.98  (0.94 to 1.03) | 1.02  (0.92 to 1.13) | 1.06  (0.95 to 1.18) | 0.92  (0.82 to 1.02) | 0.90  (0.81 to 1.00) | 0.95  (0.85 to 1.07) | 1.11  (0.97 to 1.27) | 0.91  (0.72 to 1.14) | 0.98  (0.92 to 1.04) |
| Asian # Male | 1.03  (0.96 to 1.11) | 1.06  (0.89 to 1.26) | 1.15  (0.97 to 1.37) | 0.88  (0.73 to 1.06) | 1.00  (0.84 to 1.21) | 1.01  (0.84 to 1.20) | 1.03  (0.82 to 1.28) | 1.32  (0.92 to 1.89) | 1.06  (0.96 to 1.16) |
| No database-recorded race # Male | 1.01  (0.99 to 1.04) | 0.99  (0.94 to 1.05) | 1.07*  (1.01 to 1.13) | 1.01  (0.95 to 1.07) | 0.96  (0.9 to 1.02) | 1.01  (0.95 to 1.08) | 1.02  (0.95 to 1.10) | 1.04  (0.92 to 1.17) | 0.99  (0.96 to 1.02) |
| *Race # Age category* |  |  |  |  |  |  |  |  |  |
| Black # ≥75 years | 1.00  (0.96 to 1.05) | 0.99  (0.9 to 1.11) | 1.02  (0.91 to 1.13) | 1.08  (0.97 to 1.21) | 1.02  (0.91 to 1.14) | 0.98  (0.87 to 1.09) | 0.97  (0.85 to 1.11) | 0.84  (0.67 to 1.06) | 1.00  (0.95 to 1.06) |
| Asian # ≥75 years | 0.99  (0.93 to 1.07) | 1.10  (0.93 to 1.31) | 1.02  (0.86 to 1.22) | 0.94  (0.78 to 1.13) | 1.03  (0.85 to 1.24) | 1.03  (0.86 to 1.23) | 0.77*  (0.61 to 0.97) | 1.05  (0.73 to 1.49) | 1.07  (0.98 to 1.18) |
| No database-recorded race # ≥75 years | 0.99  (0.97 to 1.02) | 1.02  (0.96 to 1.08) | 1.02  (0.96 to 1.08) | 1.04  (0.98 to 1.10) | 0.98  (0.92 to 1.04) | 1.02  (0.96 to 1.08) | 0.91  (0.85 to 0.98) | 0.89  (0.79 to 1.01) | 1.01  (0.98 to 1.04) |
| *Ethnicity # Gender* |  |  |  |  |  |  |  |  |  |
| Hispanic # Male | 1.00  (0.96 to 1.05) | 0.99  (0.89 to 1.1) | 0.94  (0.85 to 1.04) | 1.00  (0.89 to 1.11) | 1.05  (0.94 to 1.18) | 1.03  (0.92 to 1.15) | 1.05  (0.92 to 1.19) | 0.92  (0.73 to 1.17) | 1.07*  (1.01 to 1.13) |
| No database-recorded ethnicity # Male | 1.01  (0.98 to 1.03) | 1.06  (1 to 1.12) | 0.98  (0.92 to 1.04) | 0.99  (0.94 to 1.06) | 1.00  (0.94 to 1.06) | 1.02  (0.95 to 1.08) | 1.00  (0.93 to 1.08) | 1.00  (0.88 to 1.13) | **1.05****  (1.02 to 1.09) |
| *Ethnicity # Age category* |  |  |  |  |  |  |  |  |  |
| Hispanic # ≥75 years | 1.02  (0.97 to 1.06) | 1.14  (1.02 to 1.27) | 0.98  (0.88 to 1.09) | 0.94  (0.84 to 1.05) | 1.09  (0.98 to 1.22) | 0.98  (0.88 to 1.10) | 0.98  (0.85 to 1.11) | 1.02  (0.81 to 1.29) | 1.02  (0.96 to 1.08) |
| No database-recorded ethnicity # ≥75 years | 1.01  (0.99 to 1.04) | 1.02  (0.96 to 1.08) | 0.98  (0.92 to 1.04) | 0.98  (0.92 to 1.04) | 1.06  (0.99 to 1.13) | 1.00  (0.94 to 1.07) | 1.08  (1.00 to 1.16) | 1.00  (0.88 to 1.13) | 0.99  (0.96 to 1.03) |
| *Gender # Age category* |  |  |  |  |  |  |  |  |  |
| Male # ≥75 years | **1.04*****  (1.02 to 1.06) | 1.03  (0.99 to 1.08) | 1.04  (0.99 to 1.09) | 1.06*  (1.01 to 1.12) | 1.02  (0.97 to 1.07) | 1.05*  (1 to 1.11) | 1.00  (0.95 to 1.06) | 1.03  (0.94 to 1.14) | **1.04****  (1.01 to 1.07) |

^1^ In the American Geriatric Society Beers Criteria® only analyses the category “antimuscarinics” was dropped, the first-generation antihistamines and skeletal muscle relaxants categories were newly analyzed after dropping the medications from the ACB calculator, and the remaining categories remained unchanged. / ^2^ Proportion of days covered ≥80% in the 365 days prior to the index date.

Non-Bonferroni-adjusted p-values: * p<0.05, ** p<0.01, *** p<0.001; Bonferroni adjusted p-values highlighted in **bold** text: p-values ≤0.00208333 (0.05/24 comparisons)

# Table S7. Association of age, gender, ethnicity, and race and the discontinuation of high-risk medications (Cox regression model): By medication class

| **Variables** | **All medication categories** | **Anticholinergic medications** | **Central nervous system medications** | **Endocrine medications** | **Gastrointestinal medications** | **Pain medications** | **Cardiovascular medications** |
| --- | --- | --- | --- | --- | --- | --- | --- |
| **n =** | 802,475 | 106,135 | 133,635 | 47,333 | 318,783 | 160,721 | 35,868 |
| **Race (ref. White)** | HR (95% CI) | HR (95% CI) | HR (95% CI) | HR (95% CI) | HR (95% CI) | HR (95% CI) | HR (95% CI) |
| Asian | 1.06  (0.99 to 1.13) | 1.06  (0.87 to 1.3) | 1.11  (0.94 to 1.3) | 0.96  (0.71 to 1.31) | 1.12*  (1.01 to 1.24) | 0.97  (0.86 to 1.09) | 0.76  (0.47 to 1.22) |
| Black | **1.07****  (1.03 to 1.11) | 1.13*  (1.01 to 1.25) | 0.99  (0.9 to 1.09) | 1.09  (0.92 to 1.29) | 1.09**  (1.02 to 1.15) | 1.04  (0.97 to 1.11) | 1.03  (0.79 to 1.34) |
| No database-recorded race | 1.03*  (1.01 to 1.06) | 1.07  (0.99 to 1.15) | 0.99  (0.92 to 1.05) | 1.18**  (1.05 to 1.32) | 1.04  (1 to 1.08) | 1.00  (0.96 to 1.05) | 0.92  (0.77 to 1.1) |
| **Ethnicity (ref. Non-Hispanic)** |  |  |  |  |  |  |  |
| Hispanic | 1.02  (0.97 to 1.07) | 1.05  (0.93 to 1.19) | 0.95  (0.85 to 1.07) | 1.09  (0.89 to 1.34) | 1.06  (0.99 to 1.14) | 0.99  (0.9 to 1.07) | 0.97  (0.7 to 1.36) |
| No database-recorded ethnicity | 1.03*  (1.00 to 1.05) | 1.02  (0.95 to 1.08) | 0.99  (0.93 to 1.05) | 1.12*  (1.01 to 1.24) | 1.05**  (1.01 to 1.09) | 1.00  (0.95 to 1.04) | 1.17  (1 to 1.37) |
| **Gender (ref. Female)** |  |  |  |  |  |  |  |
| Male | **0.89*****  (0.87 to 0.91) | 0.93*  (0.87 to 1.00) | 0.97  (0.92 to 1.03) | 0.93  (0.84 to 1.02) | **0.92*****  (0.88 to 0.95) | **0.85*****  (0.81 to 0.88) | 0.96  (0.83 to 1.1) |
| **Age category (ref. 65-74 years)** |  |  |  |  |  |  |  |
| 75 and above | **0.86*****  (0.84 to 0.88) | **0.88*****  (0.83 to 0.94) | **0.89*****  (0.84 to 0.94) | 0.97  (0.88 to 1.07) | **0.87*****  (0.84 to 0.9) | **0.88*****  (0.84 to 0.92) | 0.86*  (0.74 to 0.98) |
| **Interaction terms** |  |  |  |  |  |  |  |
| *Race # Ethnicity* |  |  |  |  |  |  |  |
| Black # Hispanic | 0.96  (0.83 to 1.12) | 0.90  (0.58 to 1.38) | 1.05  (0.7 to 1.56) | 0.88  (0.42 to 1.83) | 0.92  (0.72 to 1.19) | 0.98  (0.73 to 1.32) | 1.49  (0.75 to 2.96) |
| Black # No database-recorded ethnicity | 0.94**  (0.90 to 0.98) | 0.90  (0.79 to 1.02) | 1.01  (0.9 to 1.14) | 0.95  (0.77 to 1.16) | 0.90**  (0.84 to 0.97) | 0.98  (0.9 to 1.07) | 1.10  (0.86 to 1.40) |
| Asian # Hispanic | 1.07  (0.89 to 1.29) | 0.96  (0.56 to 1.67) | 1.36  (0.89 to 2.07) | 2.06  (1.00 to 4.26) | 0.90  (0.66 to 1.22) | 1.02  (0.66 to 1.56) | 2.44  (0.97 to 6.12) |
| Asian # No database-recorded ethnicity | 0.96  (0.90 to 1.04) | 0.92  (0.75 to 1.15) | 0.89  (0.74 to 1.07) | 1.12  (0.81 to 1.54) | 0.94  (0.83 to 1.05) | 1.02  (0.89 to 1.18) | 1.59*  (1.04 to 2.42) |
| No database-recorded race # Hispanic | 1.01  (0.97 to 1.06) | 0.97  (0.85 to 1.12) | 1.03  (0.91 to 1.17) | 0.81*  (0.66 to 0.99) | 1.00  (0.92 to 1.08) | 1.09  (0.99 to 1.20) | 1.19  (0.89 to 1.6) |
| No database-recorded race # No database-recorded ethnicity | 0.96**  (0.94 to 0.99) | 0.97  (0.9 to 1.05) | 0.99  (0.92 to 1.06) | **0.80*****  (0.72 to 0.90) | 0.97  (0.93 to 1.01) | 0.98  (0.93 to 1.04) | 0.94  (0.8 to 1.10) |
| *Race # Gender* |  |  |  |  |  |  |  |
| Black # Male | 0.98  (0.94 to 1.03) | 0.94  (0.82 to 1.07) | 1.02  (0.91 to 1.15) | 0.87  (0.71 to 1.06) | 0.98  (0.91 to 1.05) | 0.99  (0.9 to 1.08) | 1.09  (0.84 to 1.41) |
| Asian # Male | 1.03  (0.96 to 1.11) | 0.93  (0.74 to 1.16) | 1.14  (0.95 to 1.37) | 0.95  (0.7 to 1.29) | 1.02  (0.91 to 1.15) | 1.05  (0.9 to 1.22) | 1.24  (0.8 to 1.91) |
| No database-recorded race # Male | 1.01  (0.99 to 1.04) | 1.06  (0.99 to 1.14) | 1.01  (0.95 to 1.08) | 0.99  (0.89 to 1.10) | 1.00  (0.96 to 1.04) | 1.01  (0.96 to 1.06) | 1.16  (1 to 1.34) |
| *Race # Age category* |  |  |  |  |  |  |  |
| Black # ≥75 years | 1.00  (0.96 to 1.05) | 0.98  (0.87 to 1.11) | 1.03  (0.91 to 1.16) | 0.93  (0.75 to 1.14) | 1.02  (0.95 to 1.09) | 1.00  (0.92 to 1.10) | 0.90  (0.7 to 1.15) |
| Asian # ≥75 years | 0.99  (0.93 to 1.07) | 1.01  (0.82 to 1.25) | 1.02  (0.85 to 1.23) | 0.92  (0.66 to 1.27) | 0.98  (0.87 to 1.10) | 1.06  (0.91 to 1.22) | 0.76  (0.51 to 1.15) |
| No database-recorded race # ≥75 years | 0.99  (0.97 to 1.02) | 0.93*  (0.87 to 1.00) | 1.06  (0.99 to 1.13) | 0.97  (0.87 to 1.08) | 1.01  (0.97 to 1.05) | 0.98  (0.94 to 1.04) | 0.96  (0.83 to 1.10) |
| *Ethnicity # Gender* |  |  |  |  |  |  |  |
| Hispanic # Male | 1.00  (0.96 to 1.05) | 1.05  (0.92 to 1.19) | 0.97  (0.86 to 1.09) | 1.13  (0.94 to 1.37) | 0.98  (0.91 to 1.05) | 1.01  (0.92 to 1.10) | 0.93  (0.71 to 1.22) |
| No database-recorded ethnicity # Male | 1.01  (0.98 to 1.03) | 0.95  (0.88 to 1.03) | 1.02  (0.96 to 1.09) | 1.01  (0.91 to 1.13) | 1.00  (0.96 to 1.04) | 1.04  (0.99 to 1.10) | 0.89  (0.76 to 1.03) |
| *Ethnicity # Age category* |  |  |  |  |  |  |  |
| Hispanic # ≥75 years | 1.02  (0.97 to 1.06) | 1.02  (0.9 to 1.16) | 1.03  (0.91 to 1.16) | 0.94  (0.78 to 1.15) | 1.00  (0.93 to 1.07) | 1.03  (0.94 to 1.13) | 1.04  (0.81 to 1.35) |
| No database-recorded ethnicity # ≥75 years | 1.01  (0.99 to 1.04) | 1.06  (0.99 to 1.14) | 0.98  (0.92 to 1.05) | 0.97  (0.86 to 1.08) | 1.00  (0.96 to 1.04) | 1.03  (0.98 to 1.09) | 0.97  (0.84 to 1.12) |
| *Gender # Age category* |  |  |  |  |  |  |  |
| Male # ≥75 years | **1.04*****  (1.02 to 1.06) | 1.08*  (1.02 to 1.14) | 1.02  (0.97 to 1.08) | 1.01  (0.93 to 1.10) | **1.05****  (1.02 to 1.08) | 0.99  (0.95 to 1.03) | 1.18**  (1.05 to 1.32) |

Non-Bonferroni-adjusted p-values: * p<0.05, ** p<0.01, *** p<0.001 │ Bonferroni adjusted p-values highlighted in **bold** text: p-values ≤0.00208333 (0.05/24 comparisons)
